# AMPK activation is sufficient to increase skeletal muscle glucose uptake and glycogen synthesis but is not required for contraction-mediated increases in glucose metabolism

**DOI:** 10.1016/j.heliyon.2022.e11091

**Published:** 2022-10-14

**Authors:** Ryan M. Esquejo, Bina Albuquerque, Anna Sher, Matthew Blatnik, Kyle Wald, Matthew Peloquin, Jake Delmore, Erick Kindt, Wenlin Li, Jamey D. Young, Kim Cameron, Russell A. Miller

**Affiliations:** a Internal Medicine Research Unit, Pfizer Inc., Cambridge, MA 02139, United States; b Early Clinical Development, Pfizer Inc., Groton, CT 06340, United States; c Worldwide Research, Development, and Medical Affairs, Pfizer Inc., La Jolla, CA 92037, United States; d Department of Chemical & Biomolecular Engineering, Department of Molecular Physiology & Biophysics, Vanderbilt University, Nashville, TN 37235-1604, United States; e Worldwide Research, Development, and Medical Affairs, Pfizer Inc., Cambridge, MA 02139, United States

**Keywords:** AMPK, Pharmacology, Metabolism, Skeletal muscle

## Abstract

The AMP-activated protein kinase (AMPK) is a cellular sensor of energetics and when activated in skeletal muscle during contraction can impart changes in skeletal muscle metabolism. Therapeutics that selectively activate AMPK have been developed to lower glucose levels through increased glucose disposal rates as an approach to abrogate the hyperglycemic state of diabetes; however, the metabolic fate of glucose following AMPK activation remains unclear. We have used a combination of *in vivo* evaluation of glucose homeostasis and *ex vivo* skeletal muscle incubation to systematically evaluate metabolism following pharmacological activation of AMPK with PF-739, comparing this with AMPK activation through sustained intermittent electrical stimulation of contraction. These methods to activate AMPK result in increased glucose uptake but divergent metabolism of glucose: pharmacological activation results in increased glycogen accumulation while contraction-induced glucose uptake results in increased lactate formation and glucose oxidation. These results provide additional evidence to support a role for AMPK in control of skeletal muscle metabolism and additional insight into the potential for AMPK stimulation with small molecule direct activators.

## 1 Introduction

New therapeutic approaches are needed to treat the expanding population of patients with type 2 diabetes mellitus (T2DM) around the world. One strategy to improve systemic glucose homeostasis is the restoration of normal glucose uptake into skeletal muscle, which is diminished early in the diabetic and insulin-resistant state due to impaired insulin action (Hesselink et al., 2016; Sylow et al., 2017). Numerous studies have concluded that insulin signaling and exercise induced muscle contraction are both capable of increasing skeletal muscle glucose uptake; both pathways increase glucose uptake through distinct parallel signaling that converge on an increase in plasma membrane glucose transporter 4 (GLUT4) protein (Kjobsted et al., 2018; Stanford and Goodyear, 2014; Sylow et al., 2017). There has been much interest in understanding the molecular and metabolic details of exercise induced glucose uptake, as this pathway remains intact in diabetes and could be targeted therapeutically to correct a deficit in insulin-stimulated glucose uptake.

The energy sensing AMP-activated protein kinase (AMPK) has long been suggested to play a role in exercise induced skeletal muscle signaling and glucose uptake (Mu et al., 2001; O'Neill et al., 2011; Winder and Hardie, 1996); however, this has been controversial, and some have shown AMPK is dispensable for exercise-stimulated glucose uptake (Jorgensen et al., 2004; Kjobsted et al., 2019; Lantier et al., 2014). AMPK is a heterotrimeric protein kinase that is activated both allosterically and by phosphorylation by events that change cellular energy state, such as exercise, and can then phosphorylate key proteins involved in energy consuming and producing pathways to restore cellular energy balance (Calabrese et al., 2014; Hardie, 2014). It is clear that AMPK is acutely stimulated during skeletal muscle contraction, in part through increases in metabolic demand and reductions in cellular energy charge (Birk and Wojtaszewski, 2006). Additionally, reports have been clear that AMPK activation with both direct and indirect pharmacological activators of the kinase are sufficient to increase skeletal muscle glucose uptake (Cokorinos et al., 2017; Myers et al., 2017; Zhou et al., 2001). We and others have shown that direct activators of skeletal muscle AMPK are also capable of imparting a substantial glucose lowering phenotype in non-human primates and rodents, in addition to effects throughout the body in other metabolic tissues, although it has been unclear whether the glucose lowering effects are elicited solely through skeletal muscle (Cokorinos et al., 2017; Esquejo et al., 2018; Myers et al., 2017; Salatto et al., 2017).

It remains uncertain to what extent pharmacological activation of AMPK in the skeletal muscle will emulate the beneficial metabolic state of exercised skeletal muscle. Indeed, key metabolic differences abound, not least of which is the metabolic demand that accompanies skeletal muscle contraction and surely contributes to the beneficial effects of exercise. Increasing muscle glucose uptake with pharmacological AMPK activators in the absence of increased metabolic demand may result in alterations in glucose metabolism that will be critical for the understanding of the potential therapeutic application of these agents. Using pharmacological AMPK activators and controlled skeletal muscle contraction we have performed *in vivo* and *ex vivo* studies to better detail the metabolic consequences of these two mechanisms to increase skeletal muscle glucose uptake.

## 2 Results

**Figure 1:**
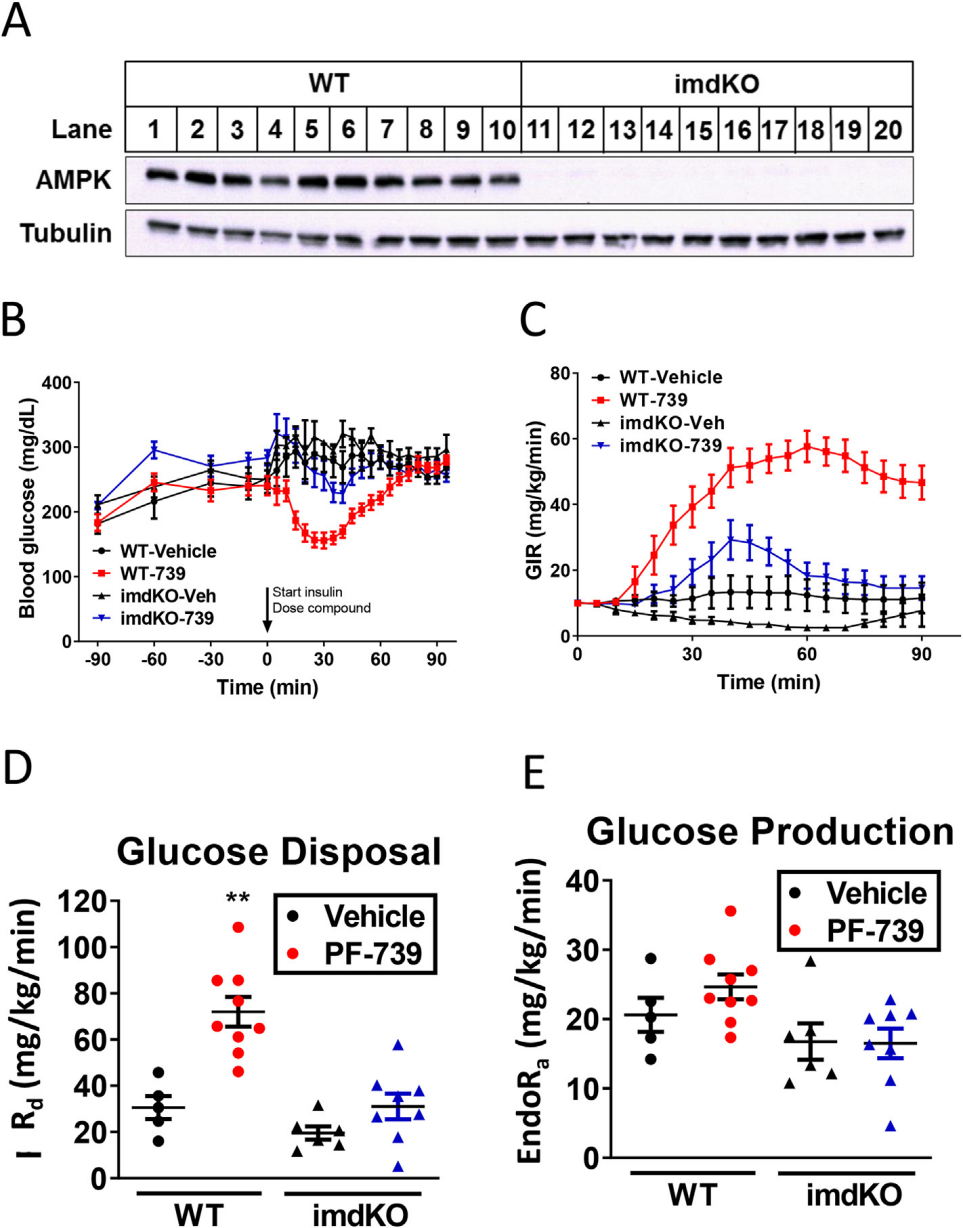
AMPK agonist PF-739 reduced blood glucose by increasing glucose disposal rate in an AMPK-dependent mechanism. A. AMPK and tubulin Western blots from gastrocnemius tissue lysates from tamoxifen-treated wild-type (wt) and inducible skeletal muscle AMPK α1/α2 knockout (imdKO) mice. B–E. Results from hyperinsulinemic-hyperglycemic clamp studies with wt and imdKO mice treated with vehicle or 100 mg/kg PF-739; (B) blood glucose levels 90 min before and 90 min after dosing insulin, vehicle or PF-739; (C) glucose infusion rate (GIR); (D) calculated glucose disposal rates; and (E) calculated glucose production. 6–8 mice were used in the study. Data are represented as mean ± SEM and analyzed using 1-way ANOVA, where ∗∗ denotes p < 0.01.

Pharmacological AMPK activation with the agonist PF-739 is able to decrease blood glucose through increases in glucose disposal rate (Cokorinos et al., 2017). We first sought to confirm that this effect was the result of increased glucose disposal in the skeletal muscle, given the potential for extra-muscle glucose uptake to contribute to glucose disposal in mice where brown fat is abundant. We generated inducible skeletal muscle-specific AMPK α1/α2 knockout mice (imdKO) that lacked AMPK alpha subunits in their muscle (Figure 1A) and dosed them subcutaneously with either vehicle or 100 mg/kg PF-739. To assess the contribution of skeletal muscle AMPK to the glucose lowering phenotype of PF-739 we performed hyperinsulinemic clamp studies with diet-induced obese (DIO) wild-type (wt) and imdKO mice subjected to vehicle or PF-739 treatment. All DIO mice were clamped to hyperglycemic levels; following vehicle or PF-739 dosing only wt animals treated with PF-739 exhibited a reduction in blood glucose that required a sustained increase in glucose infusion to maintain their glucose levels (Figure 1B-C). This effect was exclusively the result of increased glucose disposal rate, and this effect was absent in the imdKO mice (Figure 1D). There was no impact of treatment on the rate of glucose production, consistent with a skeletal muscle site of action for this glucose lowering effect (Figure 1E). This work builds on our prior studies (Cokorinos et al., 2017) and clearly establishes the skeletal muscle as the primary source for glucose lowering following acute PF-739 treatment.

**Figure 2:**
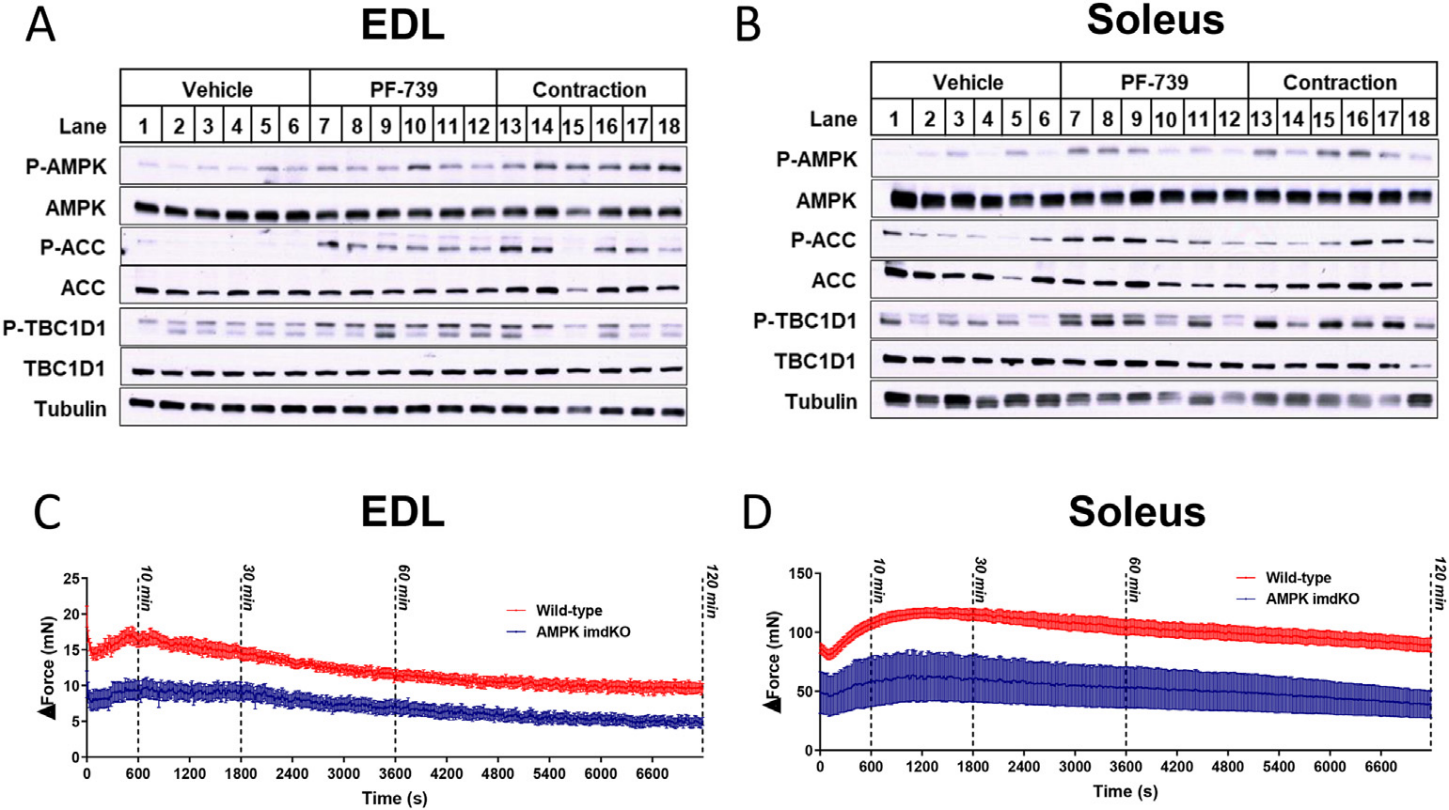
AMPK is required to maintain EDL force production during electrically stimulated muscle contraction; PF-739 and electrically stimulated muscle contraction induced AMPK signaling in extensor digitorum longus (EDL) and soleus muscle fibers *ex vivo*. Isolated EDL and soleus muscle fibers from chow fed mice were subjected to vehicle or PF-739 (10 uM) treatment, or electrically induced contraction for 30 min. Western blots for phosphorylated AMPK, ACC and TBC1D1; total AMPK, ACC, and TBC1D1; and loading control tubulin after 30 min of treatment or contraction in EDL (A) and soleus (B) muscle fibers. Force generation of electrically pulsed wt and imdKO over the course of 120 min in EDL (C) and soleus (D) muscle fibers. Data are represented as mean ± SEM.

To monitor the differences between contraction and pharmacological AMPK activation we used an *ex vivo* tissue bath that allowed for *ex vivo* electrical stimulation of small rodent muscle fibers from chow fed mice. Using both the extensor digitorum longus (EDL) and the soleus muscle we adapted a mild contraction protocol that allowed for repeated stimulation over a 2-hour period with consistent force production throughout. We measured the phosphorylation status of key proteins under these conditions and with continuous stimulation with the AMPK agonist PF-739 for a period of 30 min, revealing a similar degree of AMPK activation under both conditions as determined by the phosphorylation of AMPK and its target proteins ACC and TBC1D1 (Figure 2A-B). There was a reduction in force production in muscles obtained from imdKO mice, although the general stability of force generation was preserved for the 2-hour period (Figure 2C-D). The degree of force reduction observed in the imdKO mice was greater than that observed in recent publications of a muscle AMPK KO model where mice lack muscle AMPK from birth (Lantier et al., 2014), suggesting possibilities for muscle adaptation to AMPK deficiency.

**Figure 3:**
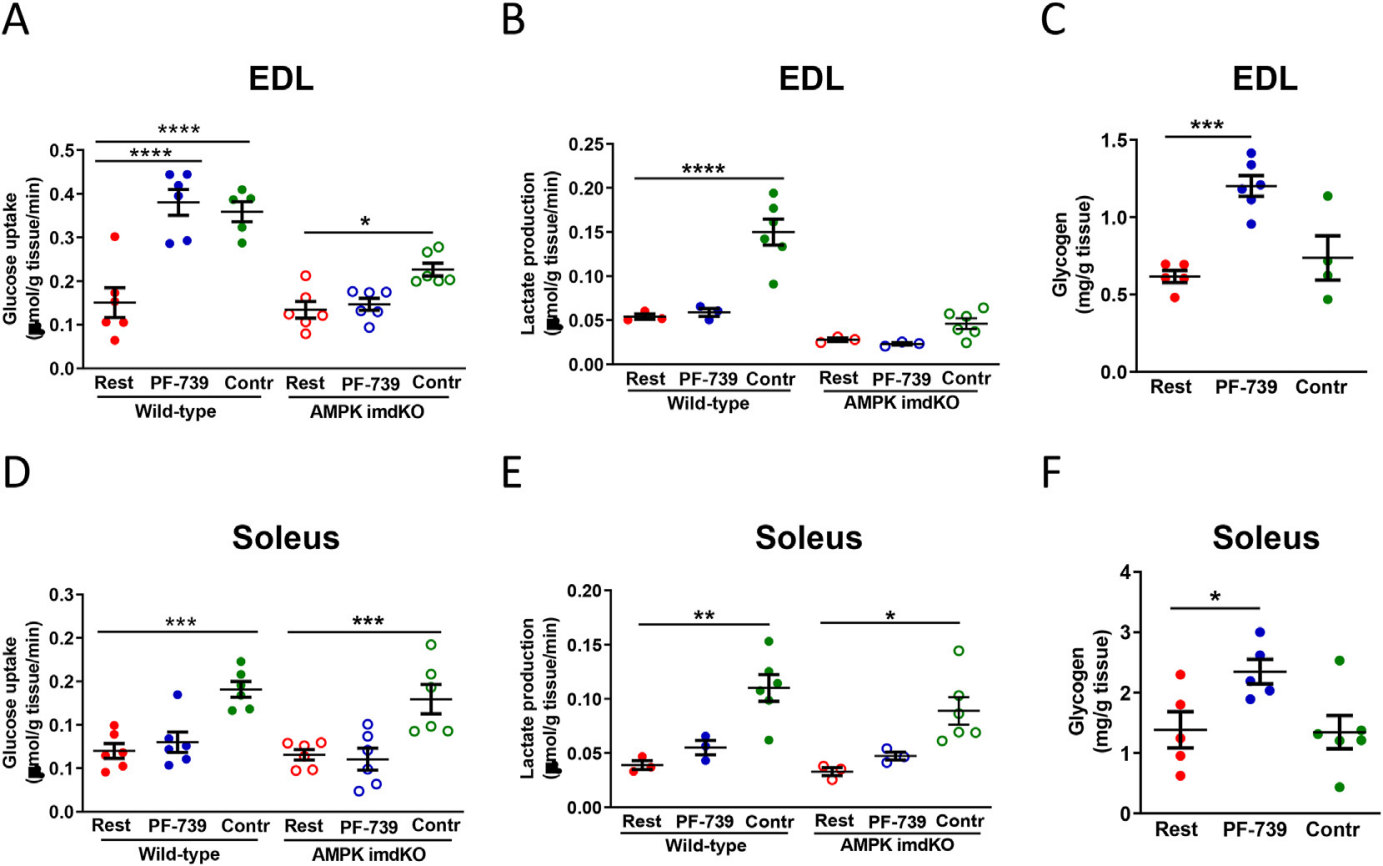
Comparing the effects of PF-739 and electrical stimulation on glucose uptake, lactate production and glycogen storage in the EDL and soleus muscle fibers *ex vivo*. A and D. Glucose uptake rate in wt and imdKO EDL (A) and soleus (D) muscle fibers from chow fed mice after being subjected to vehicle and PF-739 (10 uM) treatment, and electrical stimulation for 30 min (n = 5–6 fibers per group). B and E. Lactate production in wt and imdKO EDL (B) and soleus (E) muscle fibers lactate production when subjected to vehicle and PF-739 treatment, and electrical stimulation (n = 3–6 fibers per group). C and F. Glycogen content in EDL (C) and soleus (F) muscle fibers after vehicle or PF-739 (10 uM) treatment, and electrical stimulation for 120 min (n = 4–6 fibers per group). Contr denotes the contraction group subjected to electrical stimulation. Data are represented as mean ± SEM. 1-way ANOVA was used to evaluate statistical significance, where ∗ denotes p < 0.05, ∗∗∗ denotes p < 0.001, and ∗∗∗∗ denotes p < 0.0001.

We next evaluated both electrical stimulation and pharmacological AMPK activation on key parameters of muscle metabolism: glucose uptake rates, lactate production rates, and glycogen content. In EDL muscles from wild-type animals both PF-739 treatment and electrical stimulation produced a >2 fold increase in glucose uptake (Figure 3A). In EDL muscles collected from imdKO mice there was no effect of PF-739 treatment, consistent with a lack of AMPK and downstream signaling (Figure 3A). In contrast, electrical stimulation still resulted in a significant increase in glucose uptake, albeit a lower absolute rate of glucose uptake as compared to wt animals and a <2 fold increase compared to vehicle treated imdKO muscle (Figure 3A). In soleus muscle we observed no significant increase in glucose uptake following PF-739 treatment, but contraction still elicited an increase in glucose uptake that was not impacted by a lack of AMPK signaling (Figure 3D). We also quantified the rates of lactate export after 2 h of treatment from the muscle as this is a key molecular fate of glucose in the muscle. Only electrically-stimulated contraction resulted in an elevation in the lactate export, and this occurred in both wt and imdKO mice as well as EDL and soleus skeletal muscle (Figure 3B and 3E). Both EDL and soleus muscles from the imdKO mice did exhibit a reduced rate of basal lactate export as compared to muscle from wt animals (Figure 3B and 3E). We also measured the amount of glycogen in both the EDL and soleus of wt animals following 2 h of either electrical stimulation or PF-739 treatment. Only PF-739 treatment resulted in an increase in the glycogen content of both EDL and soleus muscle, with a more pronounced increase in glycogen in the EDL muscles, consistent with a larger impact of PF-739 on glucose uptake in EDL muscle (Figure 3C and 3F). It was of note that glycogen levels did not deplete following electrical stimulation as expected based on physiological studies (Hargreaves and Spriet, 2020); this may be due to the mild ischemic stress induced during the dissection process and depletion of internal glycogen stores.

**Figure 4:**
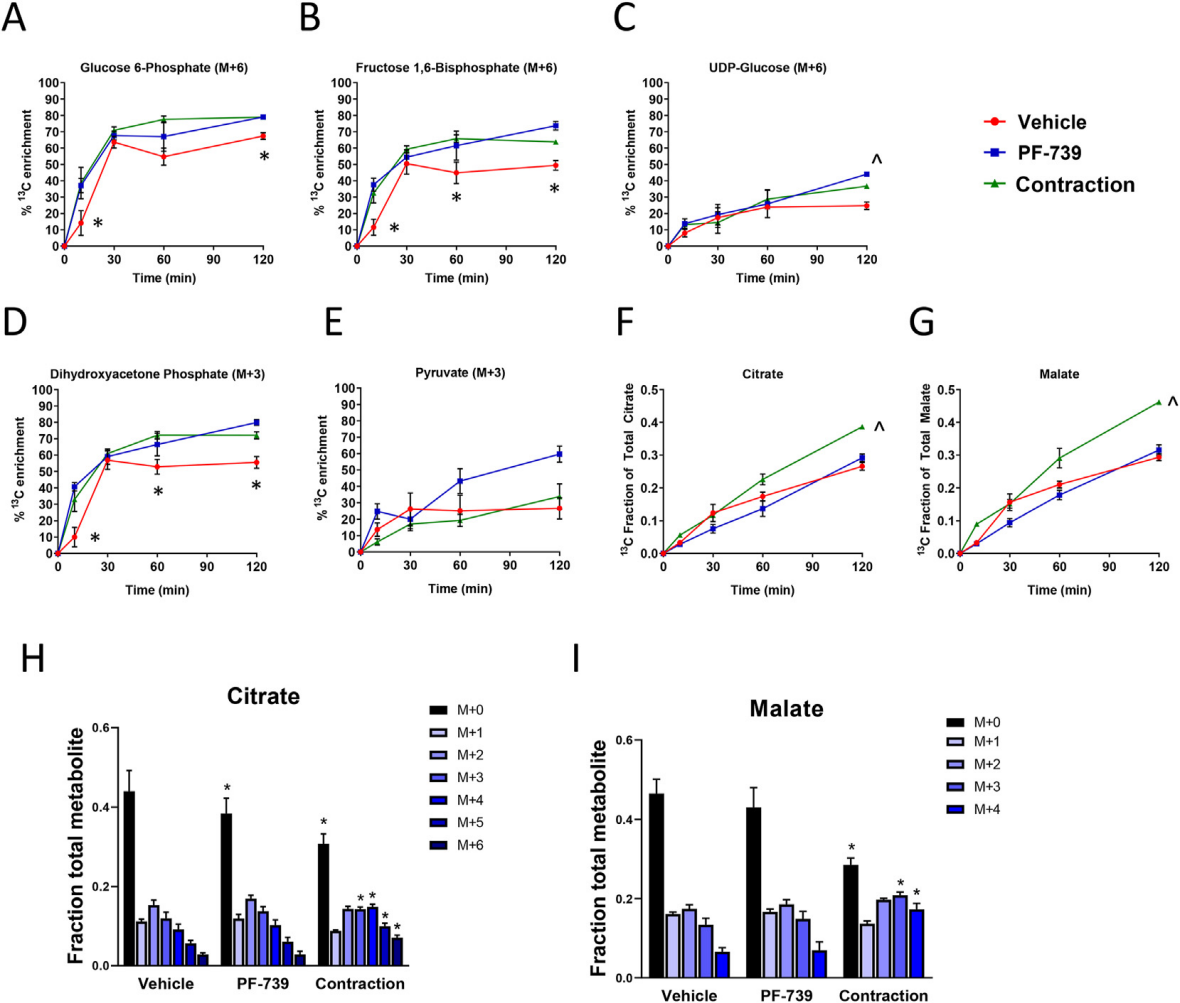
U-^13^C-glucose metabolism in EDL muscle fibers *ex vivo* from chow fed mice subjected to vehicle, PF-739 treatment, and electrically stimulated contraction. A–G. Enrichment of ^13^C-labeled glycolytic and TCA-cycle metabolites over the course of 120 min of labeling; (A) glucose 6-phosphate (M+6); (B) fructose 1,6-bisphosphate (M+6); (C) UDP-glucose (M+6); (D) dihydroxyacetone phosphate (M+3); (E) pyruvate (M+3); summation of all isotopomers and calculation of the fraction of ^13^C carbon was calculated for (F) citrate; and (G) malate. H and I. Effects of vehicle and PF-739 (PF-739) treatment and electrical stimulation on isotopomer enrichment in the EDL *ex vivo*; (H) citrate; and (I) malate, where all isotopomers (C12 = M+0; one C13 carbon = M+1, two C13 carbons = M+2; …) are reported. Contr denotes the contraction group subjected to electrical stimulation. Data are represented as mean ± SEM. ∗ denotes p < 0.05 by 2-way Anova multiple comparison test of both contraction and PF-739 with control group, and ˆ denotes p < 0.05 with the control group.

To better understand the impact on intracellular metabolism we next evaluated metabolic flux using ^13^C_6_-Glucose in the *ex vivo* tissues treated with either contraction or with PF-739. Muscle fibers were exposed to PF-739 or contraction for 15 min and then switched to a buffer containing ^13^C_6_-Glucose. We collected tissues at multiple timepoints (10, 30, 60 or 120 min) to evaluate ^13^C_6_-Glucose carbon incorporation into intracellular metabolite pools. For the glycolysis intermediates glucose-6-phosphate, fructose-1,6-bisphosphate, and dihydroxyacetone phosphate were the fully labeled species that reached steady state within 60 min; both contraction and PF-739 treatment resulted in a more rapid labeling and a significantly higher fraction of the metabolite pool fully labeled, reflecting the increase in extracellular glucose uptake (Figure 4A–E). We also measured the incorporation of ^13^C-labeled carbon derived from glucose into TCA cycle intermediates. Unlike glycolysis, labeling of the TCA cycle intermediates was slower and did not reach steady state during the 2-hour study; this is likely the result of the more complex metabolism to achieve labeling on these molecules through both labeled acetyl-CoA as well as the potential for anaplerotic pyruvate carboxylase flux (Figure 4F-G). When monitoring the fraction ^13^C-labled carbons of citrate or malate during these studies we found that contraction but not PF-739 resulted in a more rapid labeling of both TCA cycle intermediates (Figure 4F-G). This effect was also evident when examining the specific isotopologues of both citrate and malate at 2 h post treatment, suggesting a more rapid TCA cycle metabolism or a higher contribution of glucose to the TCA cycle metabolite pool (Figure 4H-I).

The labeling of pyruvate was also followed throughout the course of the experiment. In untreated muscle the pyruvate pool only achieved ∼20% labeling, despite both precursors such as dihydroxyacetone phosphate and downstream TCA cycle metabolites both achieving higher fractions of labeled carbon (Figure 4D-E). This discrepancy is likely explained by the measurement of unlabeled pyruvate that is not in equilibrium with metabolically active intracellular pyruvate, such as intratissue but extracellular pyruvate from the media that would not participate in intracellular glycolysis or subcellular compartmentalization of an unlabeled pyruvate pool that does not participate in glycolysis.

**Figure 5:**
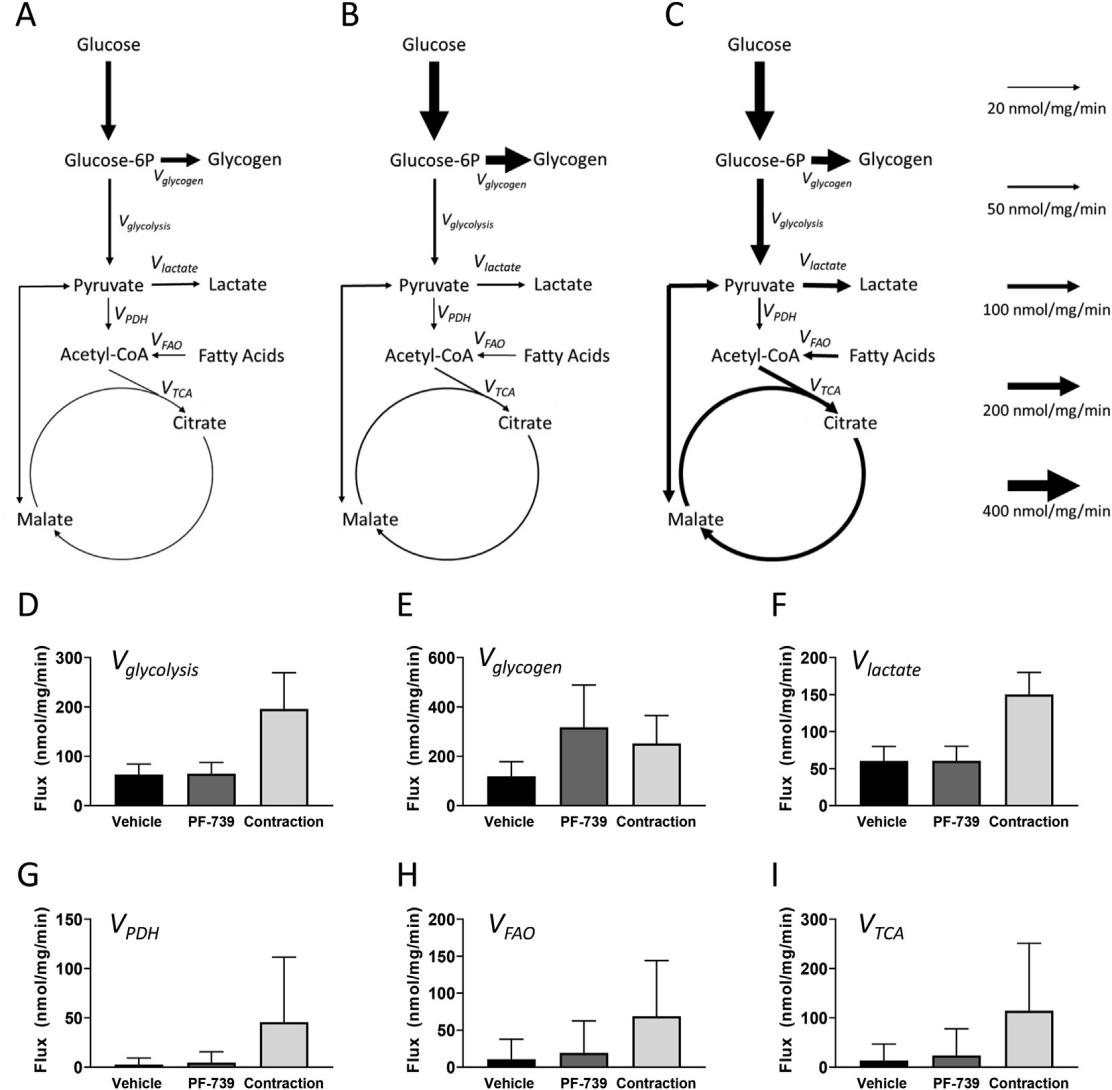
Glucose metabolic flux analysis in EDL muscle fibers *ex vivo* from chow fed mice subjected to vehicle and PF-739 treatment and electrically stimulated contraction. A–C. Models depicting glucose metabolic flux rates in EDL muscle fibers; vehicle (A), PF-739 (B) and contraction (C). D-I. Glucose metabolic flux rates in EDL muscle fibers subjected to vehicle or PF-739 (PF-‘739) treatment, or contraction. D. *V*_*glycolysis*_; E. *V*_*glycogen*_; F. *V*_*lactate*_; G. *V*_*PDH*_; H. *V*_*FAO*_; I. *V*_*TCA*_. Data are represented as mean ± SD 90% CI.

We performed isotopically non-stationary metabolic flux analysis (INST-MFA) studies of the response of EDL muscle to electrical stimulation and PF-739 treatment, integrating both the kinetic ^13^C_6_-Glucose study and the quantitative measures of glucose uptake and lactate export. We used the measured mass isotopomer distributions (MIDs), glucose uptake and lactate export data to estimate metabolic fluxes in a model of glucose metabolism in EDL. The mathematical modeling results largely recapitulated the key observations apparent in the primary data, showing a general elevation in net glycogen synthesis (i.e., the difference between glycogen deposition and degradation rates) following PF-739 treatment and predicted elevations in glycolysis, pyruvate anaplerosis and the TCA cycle fluxes following electrical stimulation (Figure 5A–F and Supplemental Figures S5-6). The model also predicted an elevation in both PDH and FAO flux following electrical stimulation, with only modest and insignificant impact on these parameters with PF-739 treatment (Figure 5G–I and Supplemental Figures S5-6).

## 3 Discussion

One of the primary metabolic defects in the insulin resistant state is an impairment in insulin-stimulated glucose uptake in the skeletal muscle; this defect in the major glucose clearing tissue has profound effects on systemic metabolism that contribute to pathology in the metabolic syndrome. To correct this impairment in insulin-stimulated glucose uptake we and others have focused on pathways that are parallel to insulin signaling to promote glucose uptake into skeletal muscle, such as the exercise-mediated glucose uptake pathway. Despite much work these pathways remain incompletely elucidated, with key controversies remaining around the role of AMPK in exercise-mediated glucose uptake. In one recent report Kjobsted and colleagues conclude that a major discrepancy in the literature is the study of the role of AMPK during or immediately after contraction (Kjobsted et al., 2019); we find this hypothesis compelling and inline with our studies here of the events that occur during contraction. Our work here has closely examined the impact of electrically-stimulated muscle contraction or direct pharmacological activation of AMPK to provide more understanding of the role AMPK plays in contraction-mediated metabolism and contextualize the potential for AMPK-activation as a therapeutic approach to the metabolic syndrome, but is limited in that we have not reported the changes in metabolism that occur after contraction, as detailed elsewhere (Kjobsted et al., 2019).

Despite multiple reports implicating AMPK as a necessary component of the exercise-induced metabolic program in muscle, we and others now conclude it is dispensable; electrically stimulated contraction remained able to increase glucose uptake and increase lactate export in the absence of any functional AMPK signaling. While we did observe that AMPK knockout tissues had modest reductions in contraction-stimulated glucose uptake and lactate export this was consistent with the reduced force generation in these tissues, suggesting that glucose uptake rate may be matched to the degree of force produced during contraction. This continues to implicate cellular mechanisms independent of AMPK whereby contraction stimulates glucose uptake acutely.

Some of the observed differences in cellular metabolism between pharmacological AMPK activation and contraction can be explained by the increased metabolic demand that is intrinsic to contraction; the ADP production during contraction is uniquely capable of driving mitochondrial metabolism and TCA induced NADH production (Chance and Connelly, 1957; Hargreaves and Spriet, 2020; Jobsis, 1963; Jobsis and Stainsby, 1968). This is consistent with our observation that contraction but not direct AMPK activation is capable of increasing TCA cycle flux. More difficult to understand is the difference in lactate export following PF-739 and contraction. Only contraction resulted in an increase in lactate export, despite similar rates of glucose uptake; more detailed studies of these differences are warranted to understand the alterations in these metabolic pathways under contraction and direct AMPK activation conditions.

Pharmacological elevations of skeletal muscle glucose uptake remains a novel approach to improve systemic metabolism in diabetic individuals. However, because of the dearth of therapeutic approaches that rely solely on this mechanism little is known about the metabolic consequences and molecular fate of elevated glucose uptake in the skeletal muscle. Our findings suggest that a major fate of glucose uptake in an *ex vivo* setting is storage in the form of intracellular glycogen. Despite this elevated glycogen synthesis, we and others have shown a robust and sustained increase in glucose uptake and plasma glucose lowering with systemic pan-AMPK activators in rodents and non-human primates (Cokorinos et al., 2017; Myers et al., 2017).

It is important to contextualize the observations in this work with the potential of AMPK activation as a therapeutic for T2DM. Increasing glucose uptake through AMPK activation is an effective approach to reduce hyperglycemia; however, it is unclear whether the physiological response to that reduction in hyperglycemia will be persistent and drive beneficial metabolic adaptation in diabetic patients. As the primary mechanism for glucose lowering is through increased glucose disposal, it is tempting to expect similar physiological adaptations to SGLT2 inhibition; however, the fate of glucose remains very different with these two mechanisms, and more work will need to be done to evaluate the consequences and alterations in the AMPK-driven glycogen accumulation we observe here. Our work here highlights a need to continue to study the long-term adaptations to AMPK activation, paying particular attention to the metabolic fate of glucose following glycogen accumulation.

In this study, we show that increasing skeletal muscle glucose uptake using an AMPK small molecule activator versus muscle contraction can lead to different metabolic fates of glucose. PF-739, a potent small molecule AMPK agonist, promotes glycogen storage; meanwhile, contraction results in less glycogen deposition and higher rates of glucose oxidation and lactate production. The differences in glucose metabolic fates between PF-739 and contraction can be likely explained by the differences in energy demand. Overall, we demonstrate glucose utilization using AMPK agonist versus contraction in an *ex vivo* skeletal muscle system.

## 4 Materials and methods

### 4.1 Mouse studies

All activities involving laboratory animals were carried out in accordance with federal, state, local and institutional guidelines governing the use of laboratory animals in research and were reviewed and approved by Pfizer Institutional Animal Care and Use Committee. AMPK *α1*^*lox/lox*^ *α2*^*lox/lox*^ *ACTA-ER2-Cre* mice on a C57Bl/6J background were purchased from Jackson Laboratories and were maintained on a 12-hour light 12-hour dark cycle and had *ad libitum* access to water and standard chow (Labdiet, PicoLab Rodent Diet 20, St. Louis, MO). Male AMPK *α1*^*lox/lox*^ *α2*^*lox/lox*^ (wild-type) and AMPK *α1*^*lox/lox*^ *α2*^*lox/lox*^ *ACTA-ER2-Cre* (AMPK imdKO) mice were dosed with tamoxifen (100 mg/kg) for 4 consecutive days. Studies were then performed 5–6 weeks after the first tamoxifen injection.

In the infusion study, diet-induced obese (DIO) male wild-type control and imdKO mice were fed a high fat diet (60% fat, Research Diets D12492) for a total of 16–20 weeks prior to study. All mice were dosed with 100 mg/kg tamoxifen for 4 consecutive days at 8–12 weeks old and implanted with an indwelling jugular vein catheter (JVC) after 8 weeks on high fat diet (all performed at Jackson Laboratories). Mice were acclimated and JVC lines flushed with heparin-saline solution. Prior to study mice were fasted overnight. At 8AM mice were connected to infusion pumps containing a 20% solution of ^13^C_6_-glucose and infusion rate of 2 μL/min was begun with animals free to move around their cage. One hour after the start of ^13^C_6_-glucose infusion animals started infusion with insulin solutions (2uM/min/kg). Thirty minutes after the start of insulin infusion blood was collected from the tail for plasma. Following blood collection animals were dosed with vehicle (20% 2-hydroxypropyl-beta-cyclodextrin (HPBCD)) or PF-739 (100 mg/kg) subcutaneously at a dosing volume of 5 mL/kg body weight. Blood glucose was measured by glucometer every 5 min and glucose was clamped to the animals starting blood glucose values to achieve the target hyperglycemic value of 250 mg/dL by altering the glucose infusion rate. Blood was collected for plasma 55 and 60 min following dosing. At the end of the study, animals were anesthetized under isoflurane while still being infused with glucose. Tissues were immediately frozen in liquid nitrogen cooled metal clamps and terminal blood samples were collected from the heart. Mice were then euthanized by cervical dislocation.

### 4.2 Mouse muscle ex vivo experiments

Extensor digitorum longus (EDL) and soleus muscle fibers were isolated from chow fed 8–12 week old male C57Bl/6J or chow fed AMPK *α1*^*lox/lox*^ *α2*^*lox/lox*^ (wild-type) and AMPK *α1*^*lox/lox*^ *α2*^*lox/lox*^ *ACTA-ER2-Cre* (AMPK imdKO) mice. Isolated muscle fibers were hung in a DMT muscle fiber myograph system (Danish Myo Technology) and incubated in an oxygenated bath filled with Buffer A (117 mM NaCl, 4.7 mM KCl, 1.2 mM MgSO_4_, 5 mM carnitine, 5 mM creatine, 1.2 mM CaCl_2_, 1.2 mM K_2_HPO_4_, 20 mM HEPES (pH 7.4), 10 mg/mL bovine serum albumin conjugated to 188 ng/mL oleic acid, 10 mM glucose). Muscle fibers were electrically pulsed at 30V, 50Hz, 1/15 s to induce contraction. All muscle fibers used in this manuscript were isolated and studied intact with no subsequent longitudinal sectioning. Supplemental figure S7 shows a timeline for all studies performed on *ex vivo* muscle fibers.

### 4.3 Western blotting

To measure AMPK signaling, EDL muscle fibers were incubated in an oxygenated Buffer A bath for 5 min. Buffer A was aspirated and replaced with fresh Buffer A containing DMSO (vehicle), PF-739 (10 μM). A subset of muscle fibers treated with vehicle were contracted by electrical pulsation for 30 min. EDL muscle fibers were stored in a tube and immediately snap frozen in liquid nitrogen. For western blotting, EDL muscle fibers were homogenized in RIPA buffer (50 mM Tris-HCl, pH 8.0, 150 mM NaCl, 1% IGEPAL, 0.5% w/v sodium deoxycholate and 0.1% w/v sodium dodecyl sulfate) with 1 mM phenylmethylsulfonyl fluoride (Thermo Fisher Scientific), protease inhibitor cocktail (Sigma) and Halt’s phosphatase inhibitor (Thermo Fisher Scientific). Protein samples were subjected to SDS-PAGE using a 4–15% gradient gel (Bio-Rad), and transferred to a nitrocellulose membrane. Membranes were incubated overnight with primary antibodies against phospho-AMPK (Thr172) (Cell Signaling, Cat# 2535S), phospho-ACC (Ser79) (Millipore, Cat#07–303), phospho-TBC1D1 (Millipore Cat#07–2268), pan-AMPKα (Cell Signaling, Cat #5831S), ACC (Cell Signaling, Cat#3662S), TBC1D1 (Cell Signaling, Cat#4629S), and α-tubulin (Cell Signaling, Cat#2125S), followed by HRP-conjugated secondary antibodies and chemiluminescent detection.

### 4.4 Glucose uptake measurement

To measure EDL glucose uptake, EDL muscle fibers were incubated in Buffer A (117 mM NaCl, 4.7 mM KCl, 1.2 mM MgSO_4_, 5 mM carnitine, 5 mM creatine, 1.2 mM CaCl_2_, 1.2 mM K_2_HPO_4_, 20 mM HEPES (pH 7.4), 10 mg/mL bovine serum albumin conjugated to 188 ng/mL oleic acid, 10 mM glucose) for 5 min and hung in DMT muscle fiber myograph system (Danish Myo Technology). Buffer was aspirated and exchanged with fresh Buffer A containing DMSO or PF-739 (10 μM); in addition, a set of vehicle-treated muscle fibers was electrically stimulated at 30 V, 50 Hz, 1/15 s for 30 min to induce muscle contraction. Buffer was replaced with Buffer A containing 500 μM 2-fluoro-2-deoxy-D-glucose (Sigma) with either DMSO or PF-739 (10 μM) for an additional 15 min. Muscle fibers were collected from the baths and excess buffer was removed by patting the muscles fibers on to an absorbent paper. Muscle fibers were stored in 2 mL Eppendorf tubed and immediately froze in liquid nitrogen. To measure glucose uptake, muscle fibers were weighed, processed and analyzed using mass spectrometry.

EDL and soleus muscle fibers were weighted and mechanically homogenized in a methanol solution. The resulting sample supernatants, along with calibration standards and quality control samples were spiked with a stable isotope labeled internal standard solution and evaporated under nitrogen gas. After reconstituting the samples with mobile phase, 4 μL was injected from each sample into the LC-MS/MS system. Adequate retention and baseline resolution of the analytes was achieved using hydrophilic interaction liquid chromatography (HILIC) with ion pairing reagents that were compatible with negative electrospray detection.

### 4.5 Lactate assay

Lactate production from EDL and soleus muscle fibers were measured using a lactate assay kit (Sigma) following the manufacturer’s protocol. *Ex vivo* muscle Buffer A was collected at the end experiment, froze in liquid nitrogen and stored in −80 °C. To measure lactate, samples of Buffer A from muscle fibers were thawed at room temperature. Samples were added to 96-well plate format in duplicates in the presence of lactate assay buffer, lactate enzyme mix and lactate probe. Samples were mixed on a plate shaker and incubated at room temperature for 30 min. Fluorescence was measured using SpectraMax M5 (Molecular Devices) plate reader (λ_excitation_ = 535/λ_emission_ = 587 nm).

### 4.6 Glycogen assay

Glycogen from EDL and soleus muscle fibers were measured using a Glycogen Colorimetric Assay kit (Abcam) following the manufacturer’s protocol. Briefly, EDL and soleus muscle fibers were homogenized in cold water and then boiled for 10 min. Homogenates were centrifuged at 18,000 × *g* at 4 °C to collect supernatant. Samples were plated in 96-well plate format in the presence or absence of hydrolysis enzyme mix. Samples were incubated at room temperature for 30 min. A mixture of Development Buffer, Development Enzyme mix and OxiRed probe was added to each well and incubated at room temperature for 30 min. Plates were read using the SpectraMax M5 (Molecular Devices) plate reader at 570 nm.

### 4.7 ^13^C-labeled glucose metabolic flux study

All metabolite measures in metabolic flux studies were made by mass spectrometry analysis. For metabolic flux studies, muscle fibers were incubated in Buffer A (117 mM NaCl, 4.7 mM KCl, 1.2 mM MgSO_4_, 5 mM carnitine, 5 mM creatine, 1.2 mM CaCl_2_, 1.2 mM K_2_HPO_4_, 20 mM HEPES (pH 7.4), 10 mg/mL bovine serum albumin conjugated to 188 ng/mL oleic acid, 10 mM glucose) for 5 min and hung in DMT muscle fiber myograph system. Buffer was aspirated and exchanged with fresh Buffer A containing DMSO or PF-739 (10 μM); in addition, a set of vehicle-treated muscle fibers was electrically stimulated at 30 V, 50 Hz, 1/15 s for 15 min to induce muscle contraction. Buffer was aspirated and muscle fibers were incubated in Buffer B (117 mM NaCl, 4.7 mM KCl, 1.2 mM MgSO_4_, 5 mM carnitine, 5 mM creatine, 1.2 mM CaCl_2_, 1.2 mM K_2_HPO_4_, 20 mM HEPES (pH 7.4), 10 mg/mL bovine serum albumin conjugated to 188 ng/mL oleic acid, 10 mM glucose U-^13^C_6_-glucose) for an additional 10, 30, 60 or 120 min and the electrical stimulation of the fibers continued. Muscle fibers were immediately removed from the baths, stored in 2 mL Eppendorf tubes and froze in liquid nitrogen. Muscle fibers were weighed, processed and analyzed using mass spectrometry.

### 4.8 Sample extraction method for mass spectrometry

Frozen, pre-weighed muscle tissue was placed in a 2.0 ml Eppendorf microcentrifuge centrifuge tube and extraction solvent (80:20 methanol:water) was added to obtain a concentration of 20 mg muscle tissue per mL extraction. Approximately 10–15 1.6 mm stainless steel beads were added to each sample tube. Samples were homogenized with a Biospec Products Minibeadbeater-96+ (Bartlesville, OK, USA) for 2 min followed by probe sonication with a Cole-Parmer 4710 series ultrasonic homogenizer (Vernon Hills, IL, USA), 40 Hz for 10 s, respectively. Homogenized material was placed in a −20 °C freezer for 30 min, then centrifuged at 14.000 rcf for 5 min. A 400 μL aliquot was transferred to a fresh 1.5 mL Eppendorf centrifuge tube and dried in a Labconco CentriVap vacuum concentrator. Samples were reconstituted with 100 ul of 95:5 Water/Methanol, vortexed and transferred to a 96-well 2 mL-polypropylene plate (Analytical Sales & Services) prior to injection.

#### 4.8.1 Negative ion LCMS

Briefly, A 5 ul aliquot was injected onto a Waters Acquity 1.8 μm, 2.1 × 100 mm HSS T3 column (Waters Corporation, Milford, MA, USA) using an Agilent 1290 Infinity II chromatography system (Agilent Technologies, Santa Clara, CA, USA) coupled to an Agilent 6545 Q-TOF mass spectrometer. Mobile phase A consisted of 95:5 water:methanol, 10 mM tributylamine, and 15 mM acetic acid. Mobile phase B was isopropyl alcohol. Analytes were eluted using a gradient of 0–5 min (0% B), 5–10 min (increase to 2% B), 10–11 min (increase to 9% B), 11–16 (hold 9% B), 16–18 min (increase to 25% B), 18–19 min (increase to 50% B), 19–25 min (hold 25% B), 25–26 min (decrease to 0% B), 26–36 min (hold 0% B). Flow rates were 0–10 min (0.400 mL/min), 10–11 min (decrease to 0.350 mL/min), 11–16 (decrease to 0.250 mL/min), 16–18 min (hold 0.250 mL/min), 18–19 min (decrease to 0.150 mL/min), 19–26 min (hold 25% B), 26–32 min (increase to 0.400 mL/min), 32–36 min (hold 0.400 mL/min). Column temperature was kept constant at 35 °C. An external standard consisting of 2-phosphoglycerate, 3-phosphoglycerate, 3-methyl-2-oxovaleric acid, α-ketoisocaproic acid, citric acid, dihydroxyacetone phosphate, dimethylallyl-pyrophosphate, fructose, fructose-1-phosphate, fructose-6-phosphate, glucose, glucose-1-phosphate, glucose-6-phosphate, glyceraldehyde-3-phosphate, glycerol phosphate, isocitric acid, isopentenyl-5-pyrophosphate, methylmalonic acid, phosphoenolpyruvate, ribose-5-phosphate, ribulose-5-phosphate, and succinic acid at approximately 45.5 μg/ml was used throughout the run to monitor for retention shifts and column performance. Reported metabolites were identified based on top level accurate mass and retention time match to authentic standards.

#### 4.8.2 Positive ion LCMS

Briefly, a 5 ul aliquot was injected onto a Waters Acquity 1.7μm 2.1 × 150mm BEH Shield C18 column using an Agilent 1290 Infinity II chromatography system coupled to a Agilent 6545 Q-TOF mass spectrometer. Mobile phase A was water containing 5 mM perfluoropentanoic acid and mobile phase B was 50:50 acetonitrile:water. Analytes were eluted at 0.400 mL/min using a gradient of 0–0.5 min (3% B), 0.5–10 min (increase to 65% B), 10–10.1 min (increase to 90% B), 10.1–12 (hold 90% B), 12–12.01 min (decrease to 25% B), 12.01–14 min (hold 50% B), 14–14.01 min (decrease to 3% B), 14.01–18 min (hold 3% B). Column temperature was held constant at 50 °C. Pierce amino acid standard H (Thermo Fisher, North America) diluted 1:100 to 0.025 umol/ml was used throughout the run to monitor retention shits and column performance. Reported metabolites were identified based on top level accurate mass and retention time match to authentic standards.

For all MS methods, the gas temperature, drying gas, nebulizer, sheath gas temperature, sheath gas flow, and VCap were set to; 325 °C, 10 L/min, 35 psig, 375 °C, 12 L/min, and −/+ 4000 V, respectively. Top level MS data was collected in positive ion mode scanning from 50 to 1700 m/z using extended linear dynamic range setting on the TOF (2 GHz). Accurate mass retention time libraries were constructed within Agilent Pathway Architect and peak integrations of targeted analytes and their isotopologues occurred within Agilent Profinder.

### 4.9 Metabolic flux analysis

The metabolic model of glucose metabolism in EDL was constructed using the INCA software package run in MATLAB 2016a (Young, 2014). The model consists of 22 biochemical reactions, 20 metabolite nodes, and 954 mass isotopomer balance equations. The model assumes (1) metabolic steady state, (2) non-stationary isotopic enrichment, (3) glucose uptake and lactate efflux constrained to measured fluxes, (4) unbalanced Glycogen pool to allow for net accumulation or depletion of glycogen, (5) equilibration between GAP and DHAP by triose phosphate isomerase, (6) presence of a cold pool for Pyruvate to account for subcellular compartmentation and incomplete pyruvate labeling, (7) rapid equilibration between MAL, FUM and OAA, (8) unlabeled carbon entry to TCA cycle via FA contribution to AcCoA which experimentally can also represent contributions from other unlabeled sources to mitochondrial PYR or AcCoA, (9) carbon cycling between PYR and MAL due to the combined activity of pyruvate carboxylase and malic enzymes described by a single reversible reaction [PYR (abc) +CO2(a) ↔ MAL (abcd)], (10) lower bounds imposed on pool sizes for metabolites G6P, F6P, F16B, PYR, LAC, AcCoA, CIT, AKG, MAL, where pool size measurements were available (Lee and Davis, 1979; Pastoris et al., 1998; Ren et al., 1993), (11) possible re-cycling or dilution of labeled CO2 formed in the reaction network by including CO2 source and sink reactions [CO_2_. source (a) → CO2 (a)] and [CO_2_ (a) → CO_2_. sink (a)]. A full description of the reaction network, isotopomer modeling approach and simulations is available in the Supplement.

Mass Isotopomer Distributions (MIDs) for the eleven measured metabolites (G6P, UDP-Glucose, F6P, F1B, DHAP, GAP, PEP, PYR, CIT, SUCC, MAL) were averaged over the six EDL muscle samples per each of the three conditions of rest, PF739 administration and contraction, and imported into INCA. The averaged MIDs were regressed using INCA to estimate the metabolic fluxes. Natural abundance of unlabeled and labeled atoms was simulated using INCA software. Relative fluxes were estimated by minimizing the sum of squared residuals (SSRs) between simulated and experimentally determined MIDs. Measurement errors were specified to be either 0.075% or the standard error of measurement, whichever was greater. Best-fit flux estimates were obtained using a minimum of 100 random initial parameter sets.

## Declarations

### Author contribution statement

Ryan Esquejo and Russell Miller: Conceived and designed the experiments; Performed the experiments; Analyzed and interpreted the data; Wrote the paper.

Bina Albuquerque: Performed the experiments; Analyzed and interpreted the data.

Anna Sher and Jamey Young: Analyzed and interpreted the data; Wrote the paper.

Matthew Blatnick; Erick Kindt; Wenlin Li and Kim Cameron: Analyzed and interpreted the data.

Kyle Wald; Matthew Peloquin and Jake Delmore: Performed the experiments.

### Funding statement

Ryan M. Esquejo was supported by 10.13039/100004319Pfizer.

### Data availability statement

Data will be made available on request.

### Declaration of interests statement

The authors declare the following conflict of interests: Some of the authors on this manuscript were employees of Pfizer Inc. during the studies reported.

### Additional information

No additional information is available for this paper.

## Appendix A Supplementary data

20220929-Esquejo-AMPKmusclemetabolism-SupplementalFigures 20220929-Esquejo-AMPKmusclemetabolism-SupplementalFigures

## References

[bib1] Birk J.B., Wojtaszewski J.F. (2006). Predominant alpha2/beta2/gamma3 AMPK activation during exercise in human skeletal muscle. J. Physiol..

[bib2] Calabrese M.F., Rajamohan F., Harris M.S., Caspers N.L., Magyar R., Withka J.M., Wang H., Borzilleri K.A., Sahasrabudhe P.V., Hoth L.R. (2014). Structural basis for AMPK activation: natural and synthetic ligands regulate kinase activity from opposite poles by different molecular mechanisms. Structure.

[bib3] Chance B., Connelly C.M. (1957). A method for the estimation of the increase in concentration of adenosine diphosphate in muscle sarcosomes following a contraction. Nature.

[bib4] Cokorinos E.C., Delmore J., Reyes A.R., Albuquerque B., Kjobsted R., Jorgensen N.O., Tran J.L., Jatkar A., Cialdea K., Esquejo R.M. (2017). Activation of skeletal muscle AMPK promotes glucose disposal and glucose lowering in non-human primates and mice. Cell Metabol..

[bib5] Esquejo R.M., Salatto C.T., Delmore J., Albuquerque B., Reyes A., Shi Y., Moccia R., Cokorinos E., Peloquin M., Monetti M. (2018). Activation of liver AMPK with PF-06409577 corrects NAFLD and lowers cholesterol in rodent and primate preclinical models. EBioMedicine.

[bib6] Hardie D.G. (2014). AMPK-sensing energy while talking to other signaling pathways. Cell Metabol..

[bib7] Hargreaves M., Spriet L.L. (2020). Skeletal muscle energy metabolism during exercise. Nat. Metabol..

[bib8] Hesselink M.K., Schrauwen-Hinderling V., Schrauwen P. (2016). Skeletal muscle mitochondria as a target to prevent or treat type 2 diabetes mellitus. Nat. Rev. Endocrinol..

[bib9] Jobsis F.F. (1963). Spectrophotometric studies on intact muscle. II. Recovery from contractile activity. J. Gen. Physiol..

[bib10] Jobsis F.F., Stainsby W.N. (1968). Oxidation of NADH during contractions of circulated mammalian skeletal muscle. Respir. Physiol..

[bib11] Jorgensen S.B., Viollet B., Andreelli F., Frosig C., Birk J.B., Schjerling P., Vaulont S., Richter E.A., Wojtaszewski J.F. (2004). Knockout of the alpha2 but not alpha1 5'-AMP-activated protein kinase isoform abolishes 5-aminoimidazole-4-carboxamide-1-beta-4-ribofuranosidebut not contraction-induced glucose uptake in skeletal muscle. J. Biol. Chem..

[bib12] Kjobsted R., Hingst J.R., Fentz J., Foretz M., Sanz M.N., Pehmoller C., Shum M., Marette A., Mounier R., Treebak J.T. (2018). AMPK in skeletal muscle function and metabolism. Faseb. J..

[bib13] Kjobsted R., Roll J.L.W., Jorgensen N.O., Birk J.B., Foretz M., Viollet B., Chadt A., Al-Hasani H., Wojtaszewski J.F.P. (2019). AMPK and TBC1D1 regulate muscle glucose uptake after, but not during, exercise and contraction. Diabetes.

[bib14] Lantier L., Fentz J., Mounier R., Leclerc J., Treebak J.T., Pehmoller C., Sanz N., Sakakibara I., Saint-Amand E., Rimbaud S. (2014). AMPK controls exercise endurance, mitochondrial oxidative capacity, and skeletal muscle integrity. Faseb. J..

[bib15] Lee S.H., Davis E.J. (1979). Carboxylation and decarboxylation reactions. Anaplerotic flux and removal of citrate cycle intermediates in skeletal muscle. J. Biol. Chem..

[bib16] Mu J., Brozinick J.T., Valladares O., Bucan M., Birnbaum M.J. (2001). A role for AMP-activated protein kinase in contraction- and hypoxia-regulated glucose transport in skeletal muscle. Mol. Cell.

[bib17] Myers R.W., Guan H.P., Ehrhart J., Petrov A., Prahalada S., Tozzo E., Yang X., Kurtz M.M., Trujillo M., Gonzalez Trotter D. (2017). Systemic pan-AMPK activator MK-8722 improves glucose homeostasis but induces cardiac hypertrophy. Science.

[bib18] O'Neill H.M., Maarbjerg S.J., Crane J.D., Jeppesen J., Jorgensen S.B., Schertzer J.D., Shyroka O., Kiens B., van Denderen B.J., Tarnopolsky M.A. (2011). AMP-activated protein kinase (AMPK) beta1beta2 muscle null mice reveal an essential role for AMPK in maintaining mitochondrial content and glucose uptake during exercise. Proc. Natl. Acad. Sci. U. S. A..

[bib19] Pastoris O., Foppa P., Catapano M., Dossena M. (1998). Metabolite concentrations in skeletal muscle of different aged rats submitted to hypoxia and pharmacological treatment with nicergoline. Exp. Gerontol..

[bib20] Ren J.M., Marshall B.A., Gulve E.A., Gao J., Johnson D.W., Holloszy J.O., Mueckler M. (1993). Evidence from transgenic mice that glucose transport is rate-limiting for glycogen deposition and glycolysis in skeletal muscle. J. Biol. Chem..

[bib21] Salatto C.T., Miller R.A., Cameron K.O., Cokorinos E., Reyes A., Ward J., Calabrese M.F., Kurumbail R.G., Rajamohan F., Kalgutkar A.S. (2017). Selective activation of AMPK beta1-containing isoforms improves kidney function in a rat model of diabetic nephropathy. J. Pharmacol. Exp. Therapeut..

[bib22] Stanford K.I., Goodyear L.J. (2014). Exercise and type 2 diabetes: molecular mechanisms regulating glucose uptake in skeletal muscle. Adv. Physiol. Educ..

[bib23] Sylow L., Kleinert M., Richter E.A., Jensen T.E. (2017). Exercise-stimulated glucose uptake - regulation and implications for glycaemic control. Nat. Rev. Endocrinol..

[bib24] Winder W.W., Hardie D.G. (1996). Inactivation of acetyl-CoA carboxylase and activation of AMP-activated protein kinase in muscle during exercise. Am. J. Physiol..

[bib25] Young J.D. (2014). INCA: a computational platform for isotopically non-stationary metabolic flux analysis. Bioinformatics.

[bib26] Zhou G., Myers R., Li Y., Chen Y., Shen X., Fenyk-Melody J., Wu M., Ventre J., Doebber T., Fujii N. (2001). Role of AMP-activated protein kinase in mechanism of metformin action. J. Clin. Invest..

