# Supplementary material for: AMPK activation is sufficient to increase skeletal muscle glucose uptake and glycogen synthesis but is not required for contraction-mediated increases in glucose metabolism
Source: Heliyon. 2022 Oct 14;8(10):e11091. doi: 10.1016/j.heliyon.2022.e11091 (PMC9593205; doi:10.1016/j.heliyon.2022.e11091)
Supplement: 20220929-Esquejo-AMPKmusclemetabolism-SupplementalFigures [file mmc1.docx]

**Supplemental Figure 1. Metabolic mathematical model of glucose metabolism in EDL.**
A. Diagram of the complete reaction network with the measured metabolites and fluxes highlighted in orange. B. List of biochemical reaction equations. C. Model input fluxes and pools.

Supplemental Figure 1


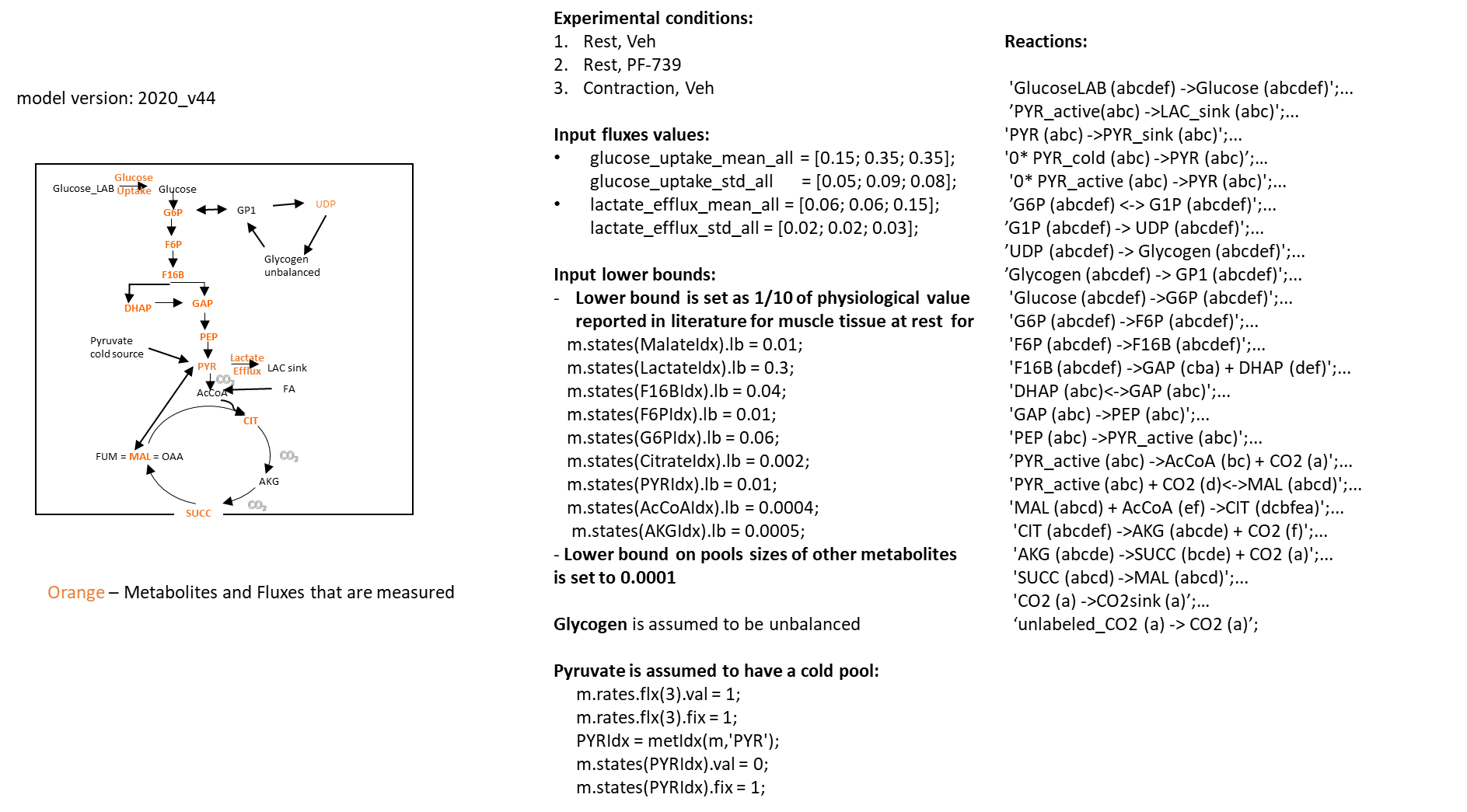

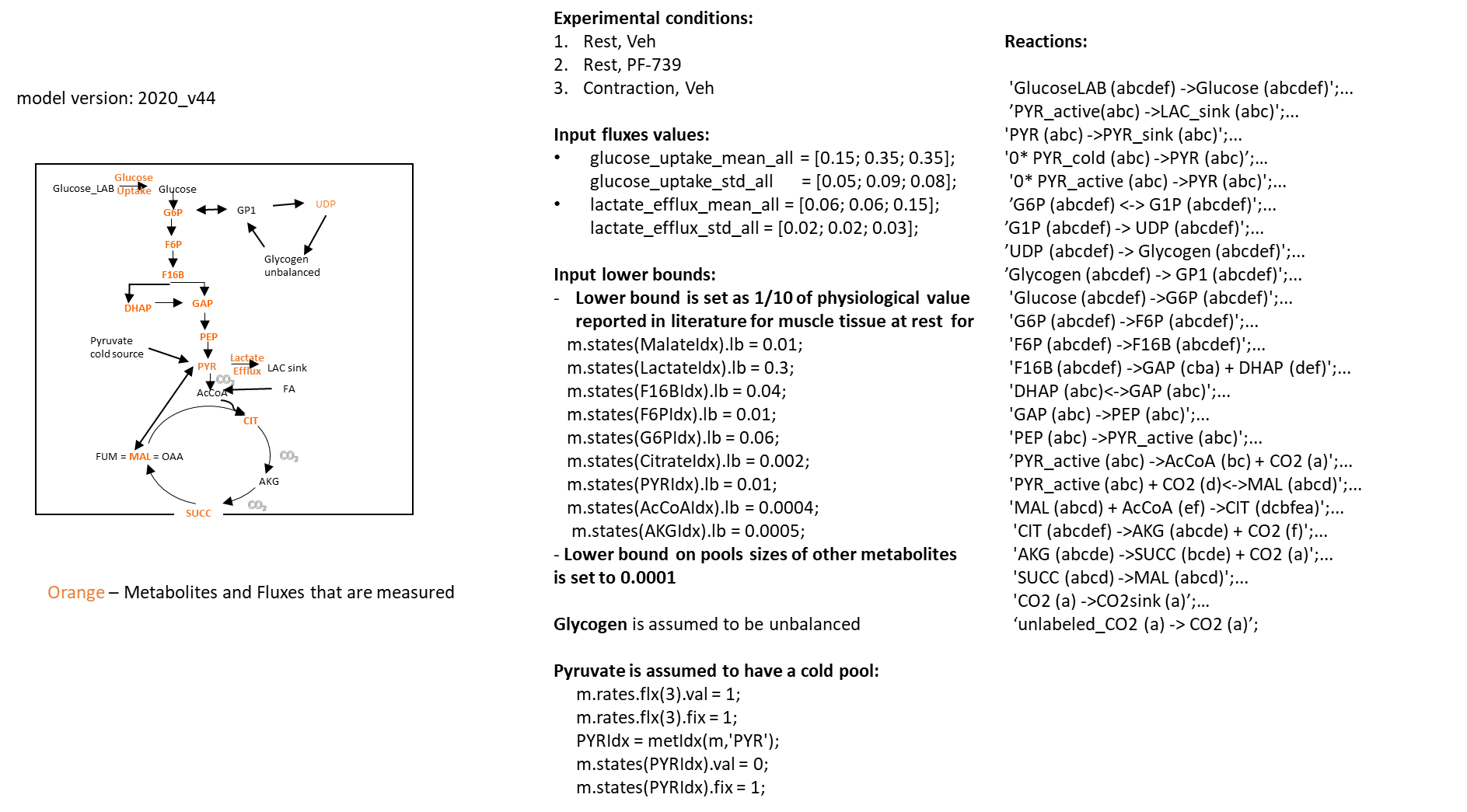


B

A


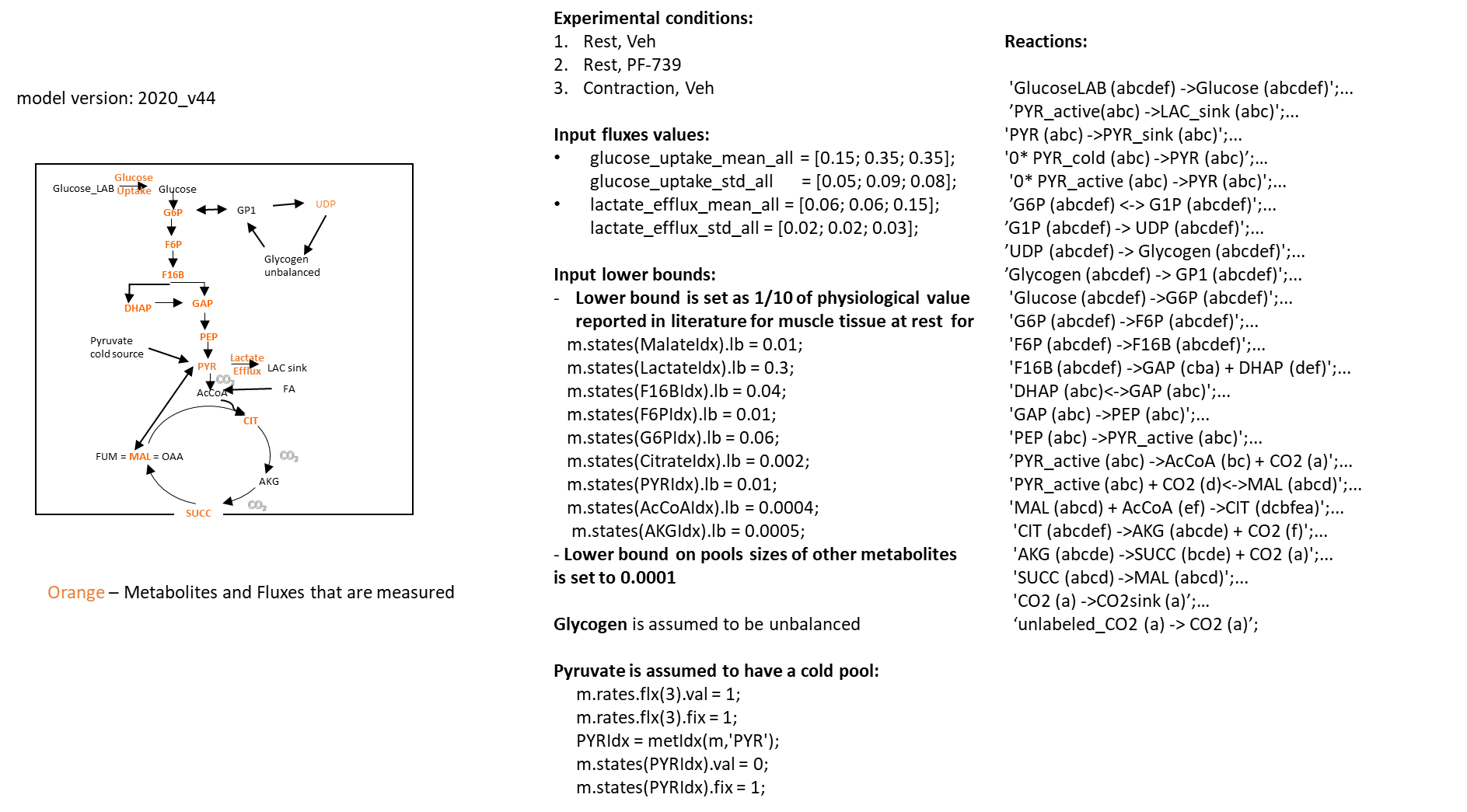


C

**Supplemental Figure 2. Best-fit model simulated MIDs compared to experimentally measured MIDs determined under rest condition.**

Best-fit model simulation results of MID time courses (solid lines) compared to measured MID data (mean - circle, std - error bar) for eleven measured metabolites (G6P, UDP-Glucose, F6P, F1B, DHAP, GAP, PEP, PYR, CIT, SUCC, MAL) under rest. The Mn values represent the fractional abundance of mass isotopomers with n heavy atoms.


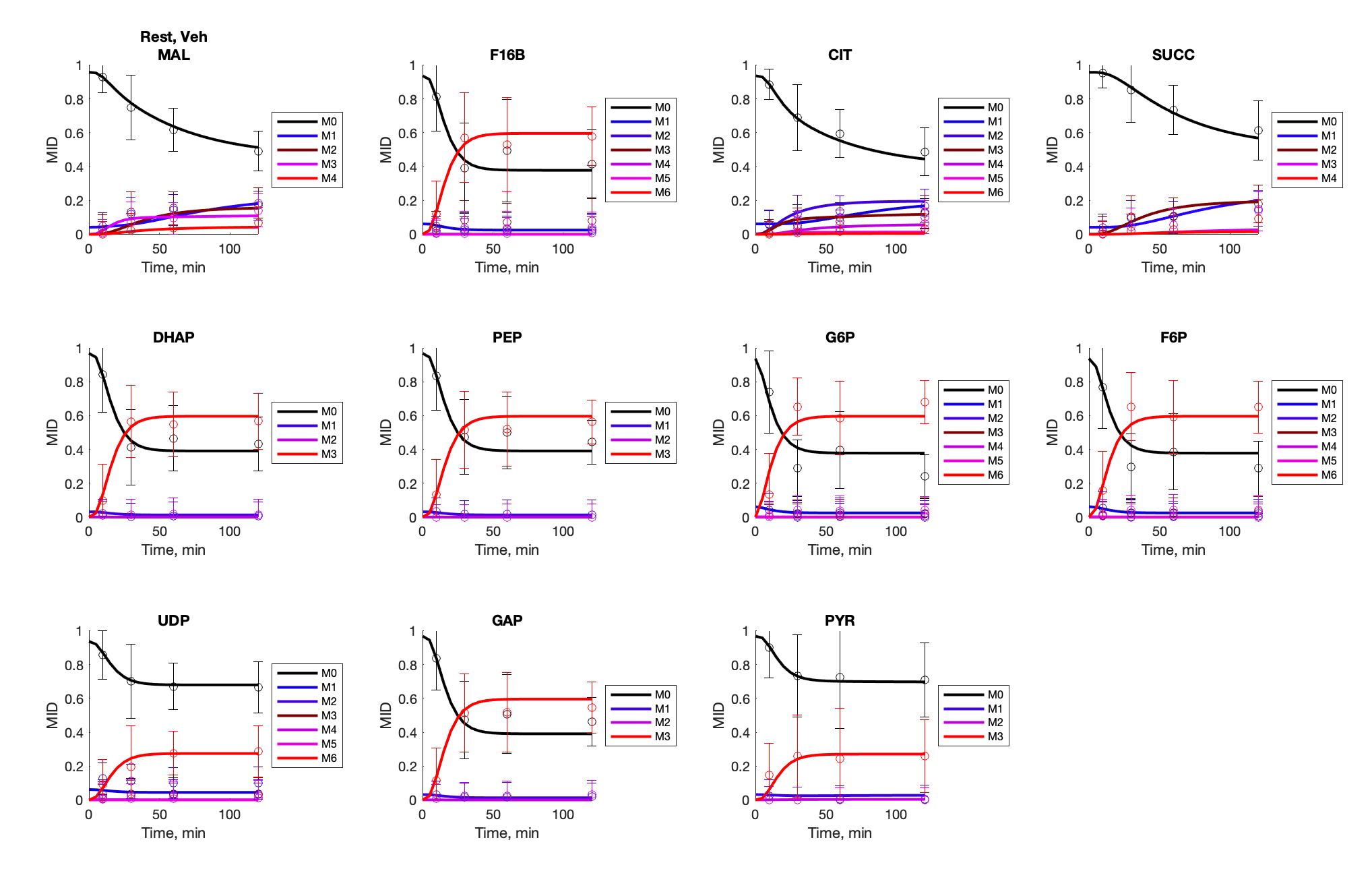


Supplemental Figure 2

**Supplemental Figure 3. Best-fit model simulated MIDs compared to experimentally measured MIDs determined under PF739 administration.**

Best-fit model simulation results of MID time courses (solid lines) compared to measured MID data (mean - circle, std - error bar) for eleven measured metabolites (G6P, UDP-Glucose, F6P, F1B, DHAP, GAP, PEP, PYR, CIT, SUCC, MAL) under PF739 administration. The Mn values represent the fractional abundance of mass isotopomers with n heavy atoms.


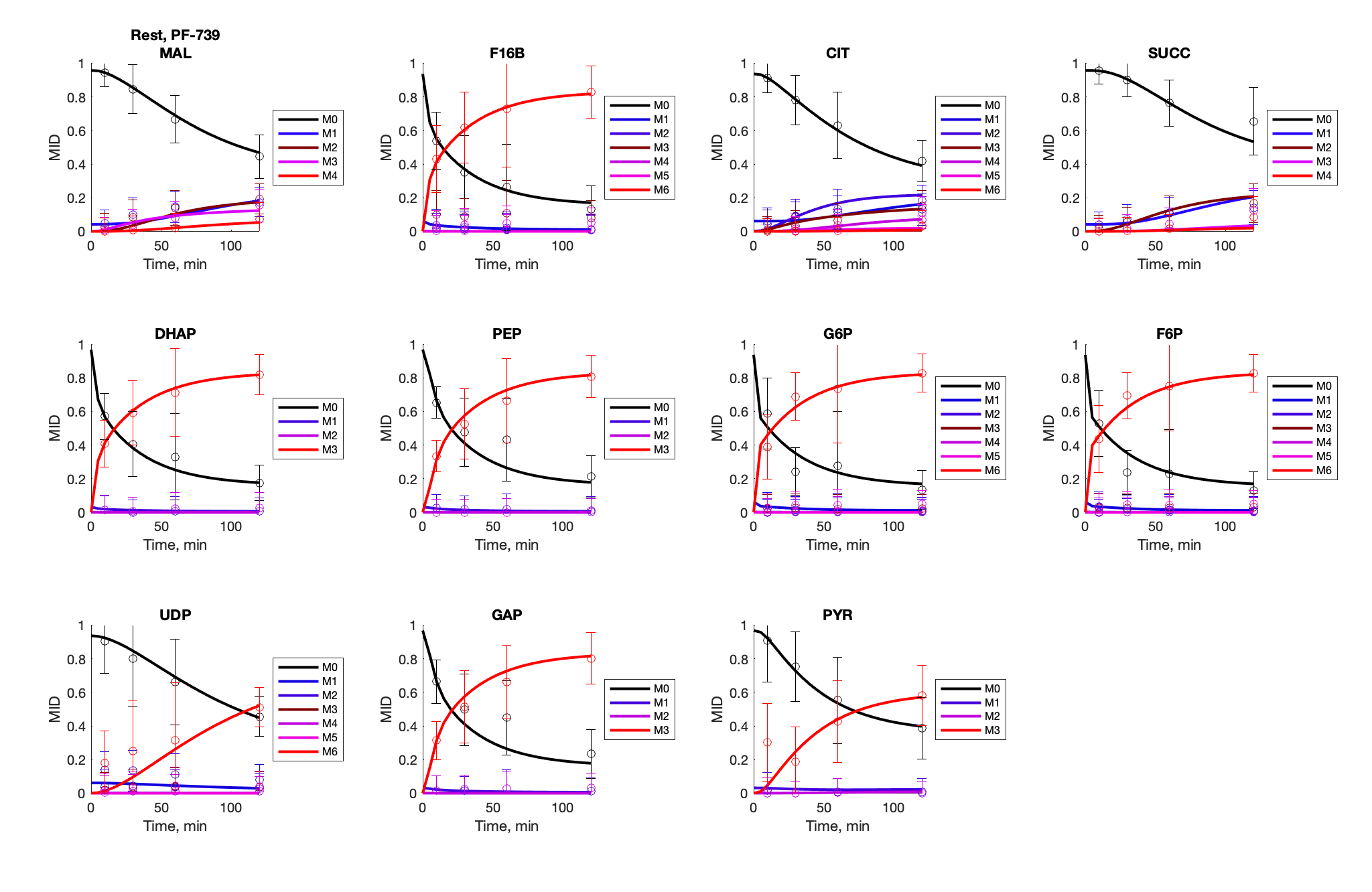


Supplemental Figure 3

**Supplemental Figure 4. Best-fit model simulated MIDs compared to experimentally measured MIDs determined under contraction condition.**

Best-fit model simulation results of MID time courses (solid lines) compared to measured MID data (mean - circle, std - error bar) for eleven measured metabolites (G6P, UDP-Glucose, F6P, F1B, DHAP, GAP, PEP, PYR, CIT, SUCC, MAL) under contraction. The Mn values represent the fractional abundance of mass isotopomers with n heavy atoms.


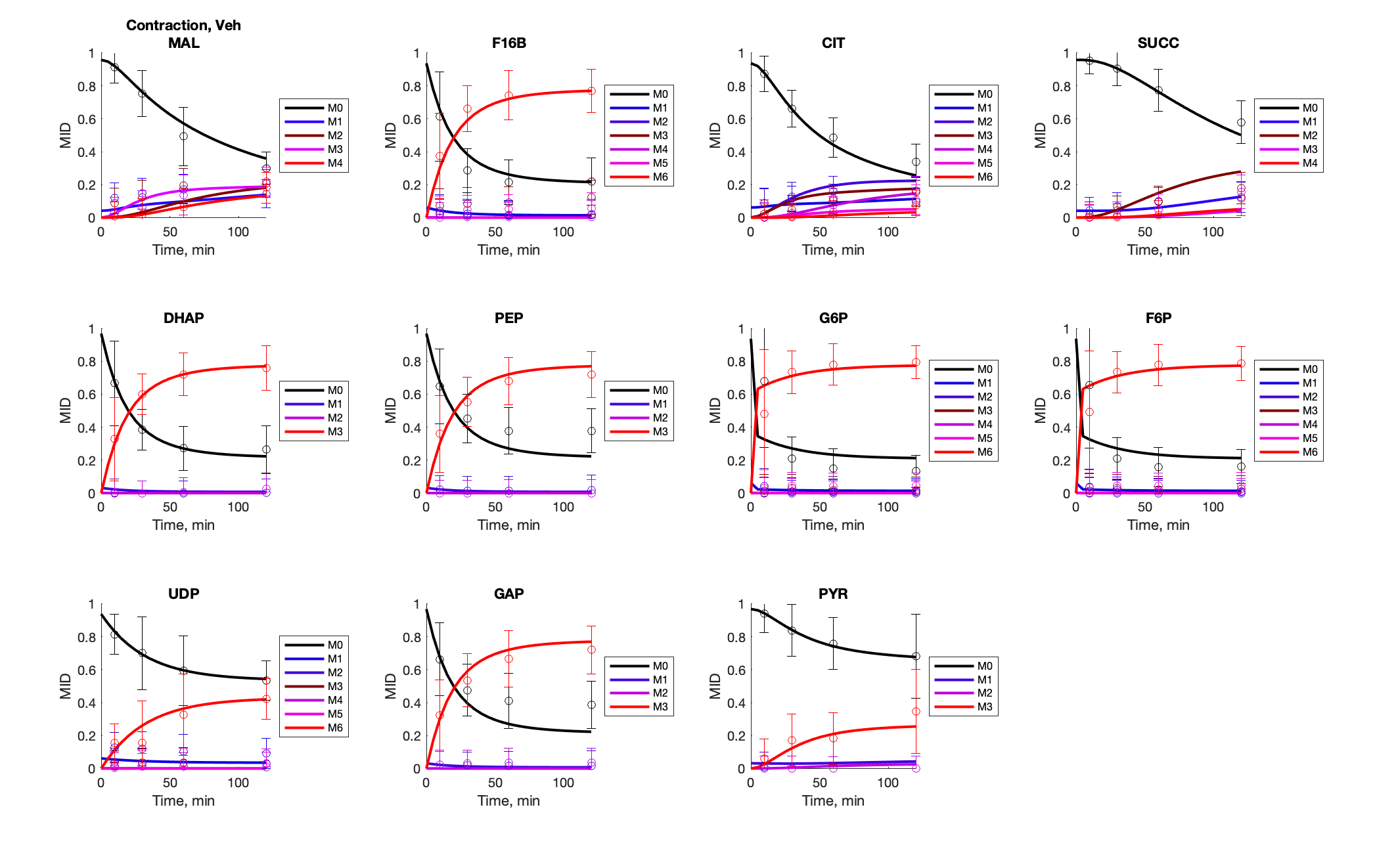


Supplemental Figure 4

**Supplemental Figure 5. Net and exchange INST-MFA flux estimates.**Best-fit model-estimated net and exchange fluxes (mean and std) for all biochemical network reactions under conditions of rest (Flux 1, Std 1), PF739 administration (Flux 2, Std 2), and contraction (Flux 3, Std 3).


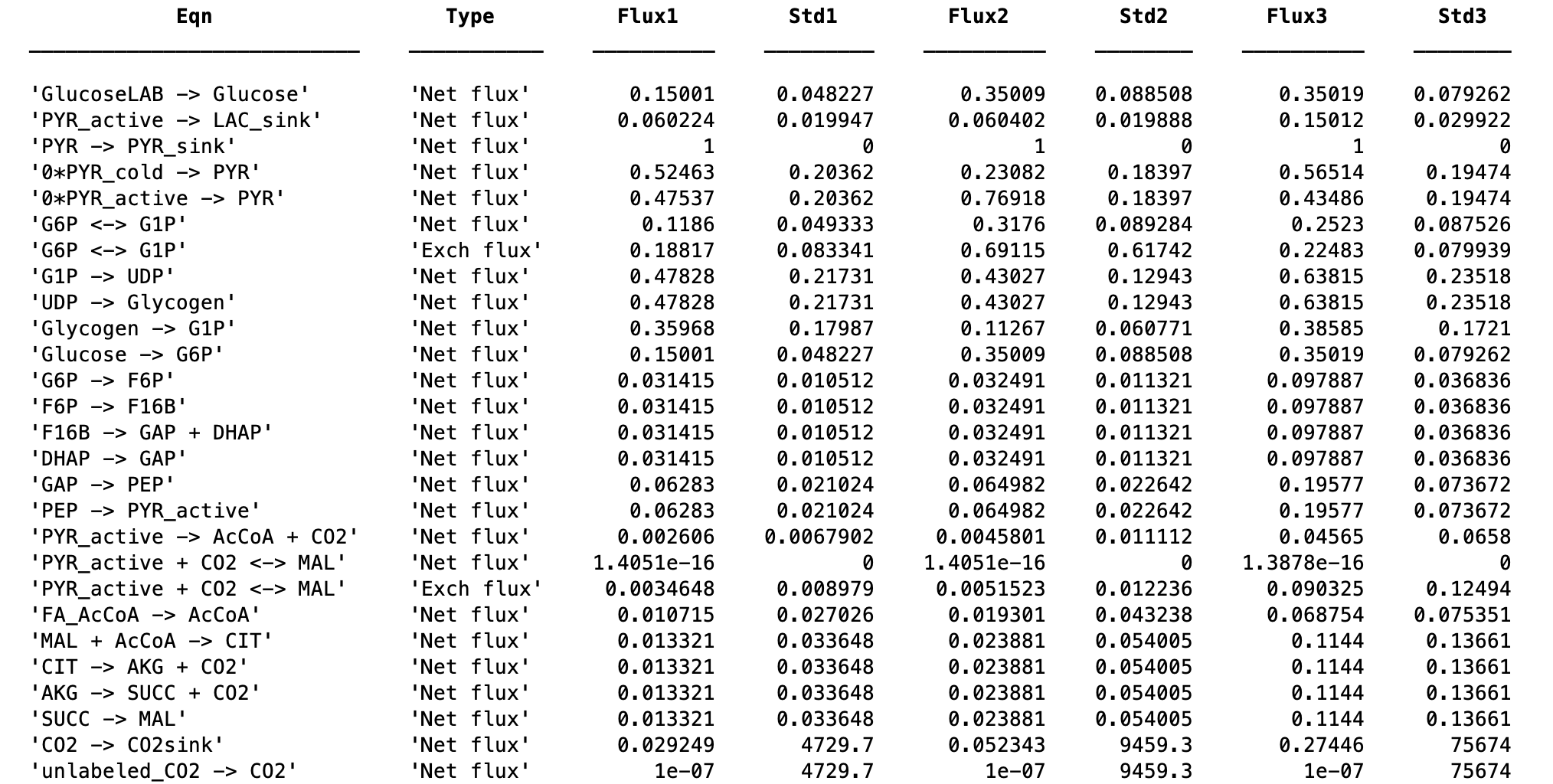


Supplemental Figure 5


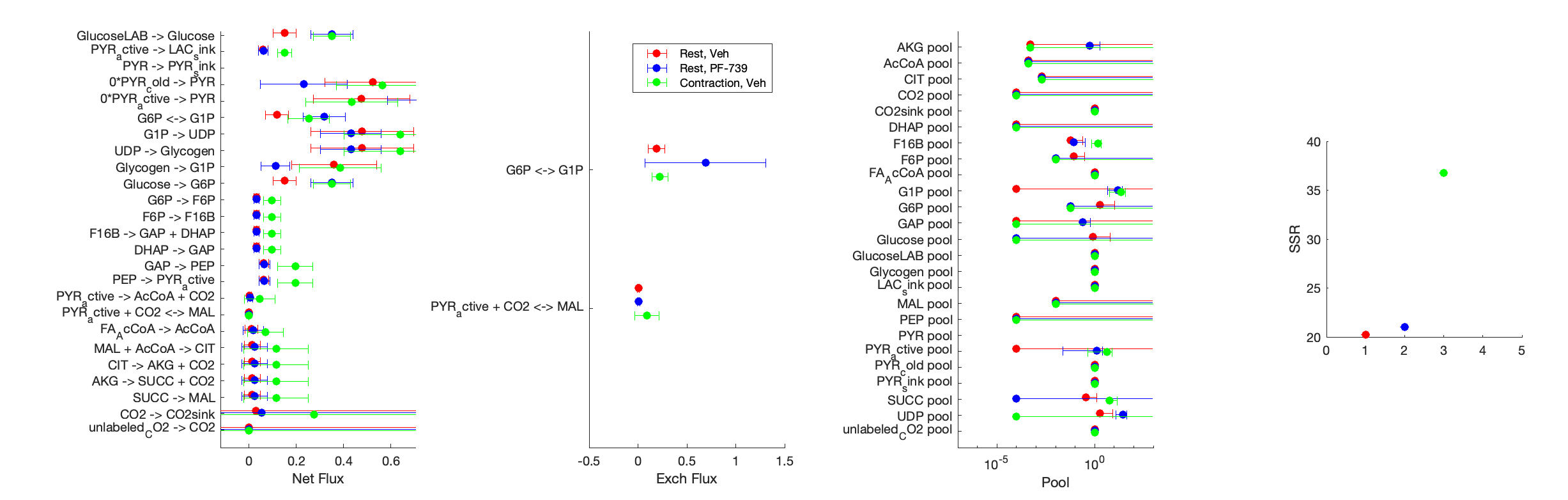

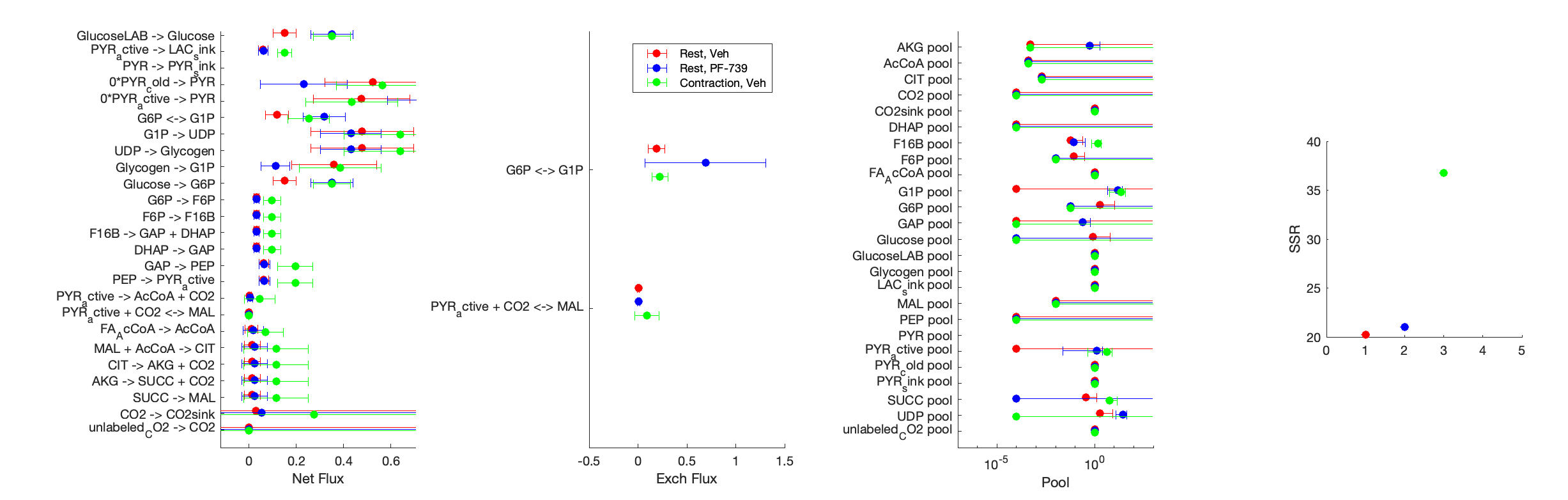
**Supplemental Figure 6. Comparison of model fits for each treatment condition.**Best-fit model-estimated net (A) and exchange (B) fluxes, pool sizes (C), and the sum of squared residuals (SSR) compared to the expected range determined from the corresponding chi-square cumulative distribution function (D) under conditions of rest (red), PF739 administration (blue), and contraction (green).

B

A


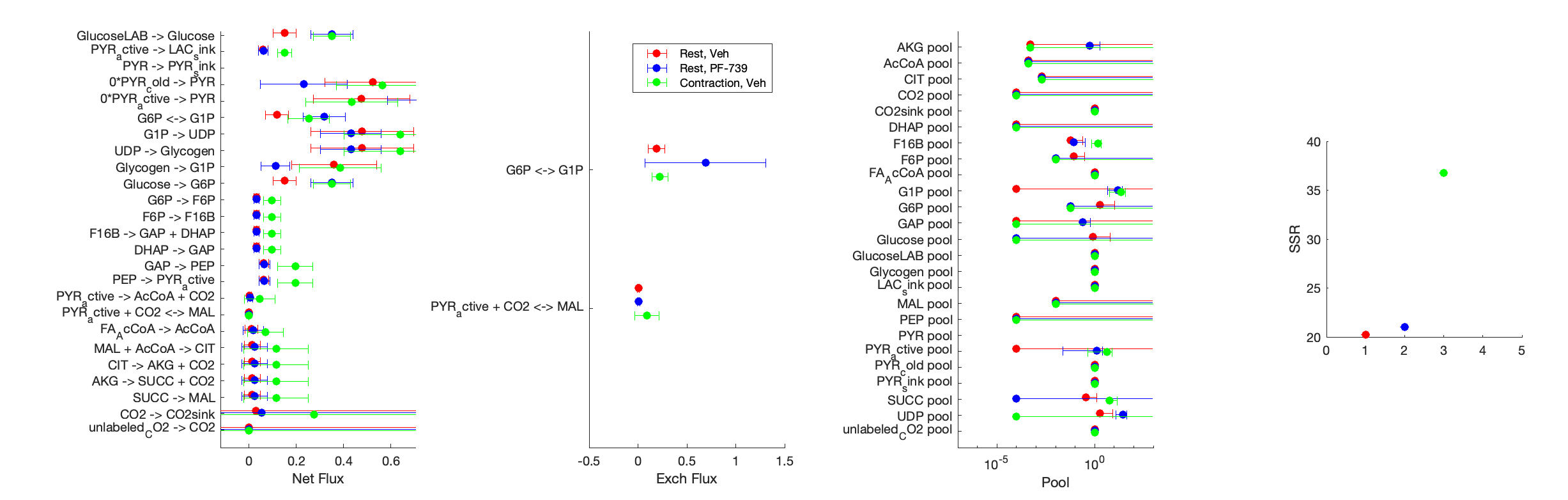

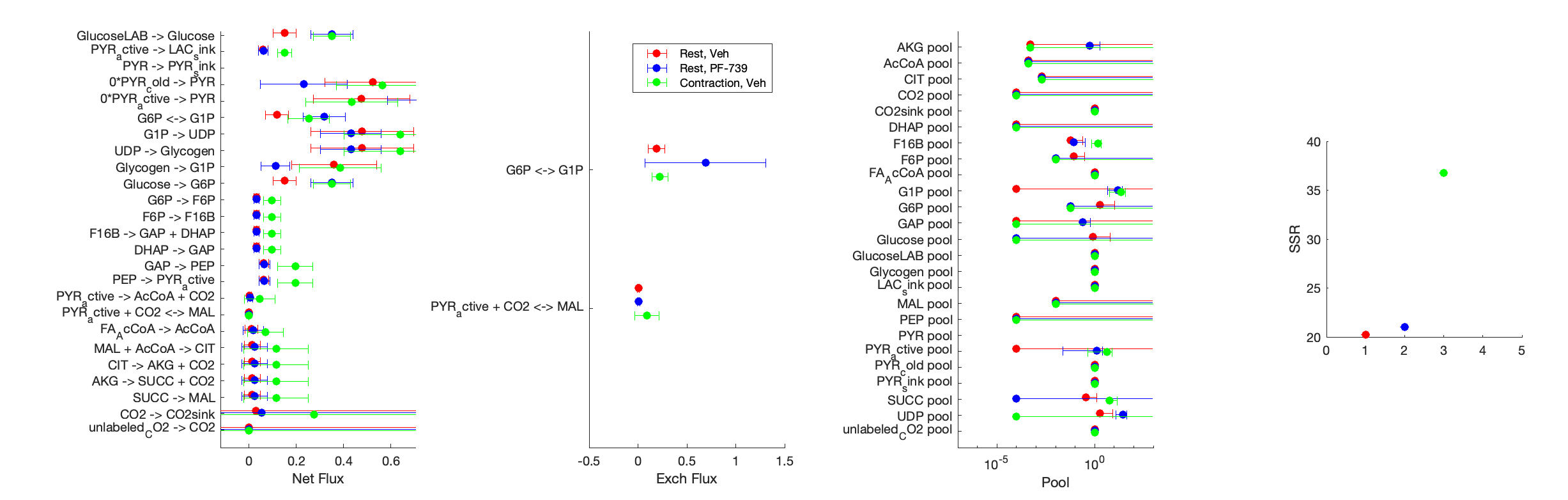


C

D

**Supplemental Figure 7. Overview of the timing for ex vivo muscle fiber studies.**

-5

0

Bath

incubation

Treatment or

contraction

30

Time (min):

A) *Western blotting*

-5

0

Bath

incubation

30

Time (min):

B) *Glucose uptake and lactate measurement*

45

Lactate

measurement

-5

0

Bath

incubation

Treatment or contraction

25

Time (min):

C) *^13^C-labeled Glucose Metabolic Flux Study*

45

^13C-glucose^

15

75

135

Collect fibers

Treatment or contraction

2-fluoro-2-deoxy-D-glucose

Supplemental Figure 7

**Supplemental Figure 8. Uncropped western blot images**


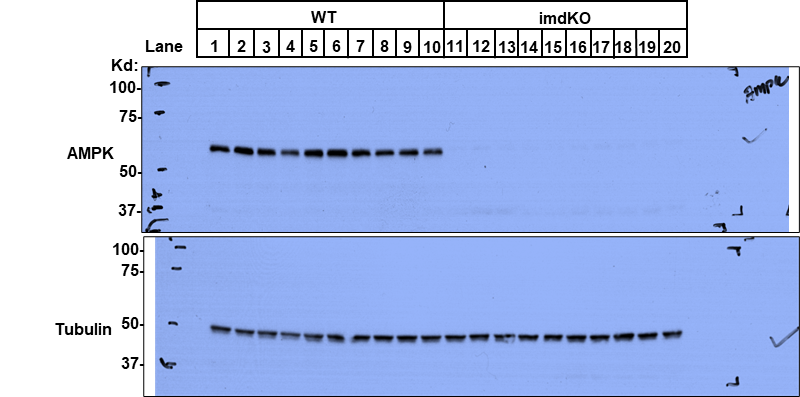


Figure 1

A

**Supplemental Figure 9. Uncropped western blot images**


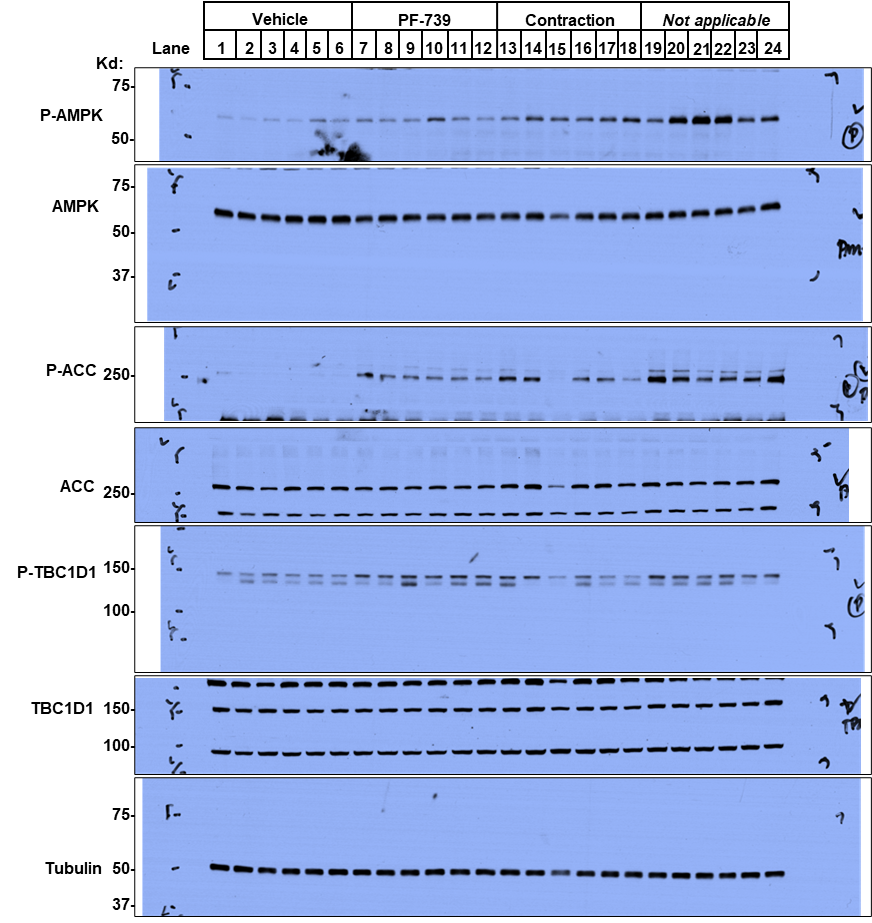


A

**EDL**

Figure 2

**Supplemental Figure 10. Uncropped western blot images**


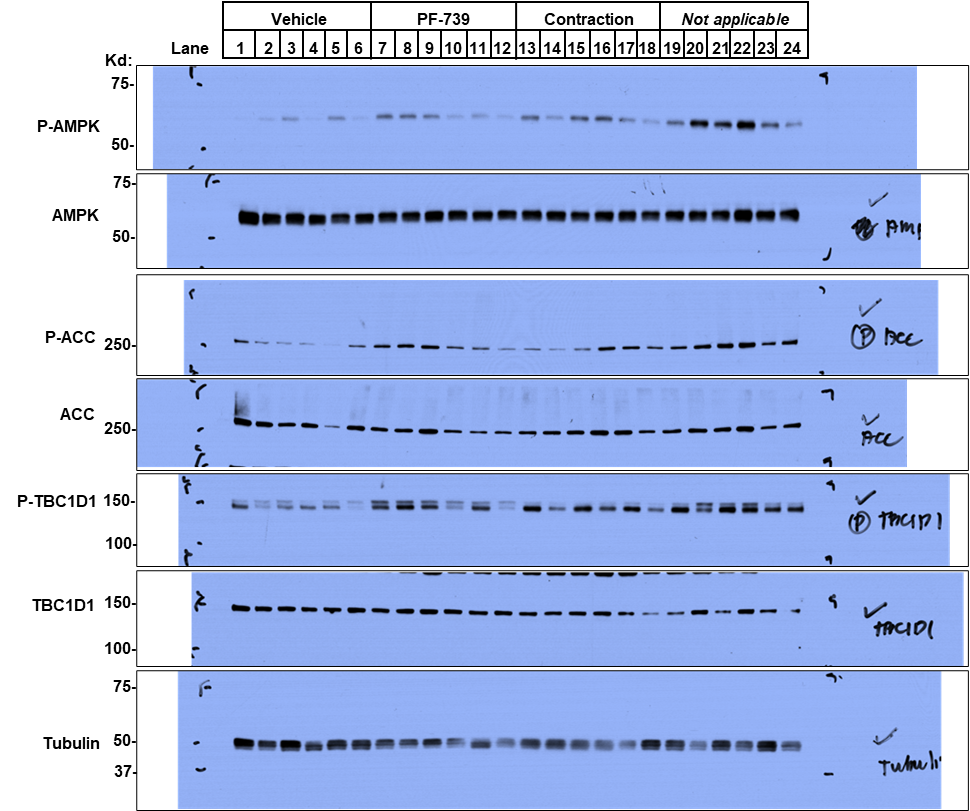


B

**Soleus**

Figure 2
